# Burden of disease due to amphetamines, cannabis, cocaine, and opioid use disorders in South America, 1990–2019: a systematic analysis of the Global Burden of Disease Study 2019

**DOI:** 10.1016/S2215-0366(22)00339-X

**Published:** 2023-02

**Authors:** João M Castaldelli-Maia, Yuan-Pang Wang, Andre R Brunoni, Andre Faro, Rafael A Guimarães, Giancarlo Lucchetti, Miquel Martorell, Rafael S Moreira, Kevin Pacheco-Barrios, Jefferson A B Rodriguez, Leonardo Roever, Diego A S Silva, Marcos R Tovani-Palone, Pascual R Valdez, Ivan R Zimmermann, Garland T Culbreth, Simon I Hay, Christopher J L Murray, Isabela M Bensenor

**Affiliations:** a Department of Psychiatry, University of São Paulo, São Paulo, Brazil; b Department of Internal Medicine, University of São Paulo, São Paulo, Brazil; c Department of Epidemiology, Mailman School of Public Health, Columbia University, New York, NY, USA; d Department of Medical Psychology, School of Medical Sciences, National University of Asuncion, San Lorenzo, Paraguay; e Department of Neuroscience, Medical School, FMABC University Centre, Santo André, Brazil; f Department of Psychology, Federal University of Sergipe, São Cristóvão, Brazil; g Faculty of Nursing, Federal University of Goiás, Goiânia, Brazil; h School of Medicine, Federal University of Juiz de Fora, Juiz de Fora, Brazil; i Department of Nutrition and Dietetics, University of Concepción, Concepción, Chile; j Centre for Healthy Living, University of Concepción, Concepción, Chile; k Department of Public Health, Oswaldo Cruz Foundation, Federal University of Pernambuco, Recife, Brazil; l Physical Medicine and Rehabilitation, Spaulding Rehabilitation Hospital, Harvard University, Boston, MA, USA; m Vicerrectorado de Investigacion, Universidad San Ignacio de Loyola, Lima, Peru; n Department of Pharmacology and Toxicology, University of Antioquia, Medellin, Colombia; o Department of Clinical Research, Federal University of Uberlândia, Uberlândia, Brazil; p Department of Physical Education, Federal University of Santa Catarina, Florianópolis, Brazil; q Saveetha Dental College and Hospitals, Saveetha Institute of Medical and Technical Sciences, Chennai, India; r Modestum, Eastbourne, UK; s Argentine Society of Medicine, Buenos Aires, Argentina; t Velez Sarsfield Hospital, Buenos Aires, Argentina; u Departamento de Saúde Coletiva, Brasília University, Brasília, Brazil; v Institute for Health Metrics and Evaluation, Seattle, WA, USA; w Department of Health Metrics Sciences, School of Medicine, University of Washington, Seattle, WA, USA

## Abstract

**Background:**

South America's substance use profile, poverty, income inequality, and cocaine-supplier role make it a unique place for substance use research. This study investigated the burden of disease attributable to amphetamine use disorder, cannabis use disorder (CAD), cocaine use disorder, and opioid use disorder (OUD) in South America from 1990 to 2019, on the basis of the Global Burden of Diseases, Injuries, and Risk Factors Study (GBD) 2019.

**Methods:**

GBD 2019 estimated the incidence, prevalence, mortality, years of life lost (YLL), years of life lived with disability (YLD), and disability-adjusted life-years (DALYs) due to substance use disorders in each of the 12 South American countries (Argentina, Bolivia, Brazil, Chile, Colombia, Ecuador, Guyana, Paraguay, Peru, Suriname, Uruguay, and Venezuela). Data were modelled using standardised tools (ie, the Cause of Death Ensemble model, spatio-temporal Gaussian process regression, and disease modelling meta-regression) to generate estimates of each quantity of interest by sex, location, and year. The analysis included comparisons by sex and country, and against regional and global estimates.

**Findings:**

In 2019, the highest amphetamine use disorder burden per 100 000 population in South America was in Peru (66 DALYs). CAD DALY rates per 100 000 in South America were stable between 1990 and 2019, except in Chile and Colombia, which had the highest rates in 2019 (19 DALYs for Chile and 18 DALYs for Colombia). OUD DALYs per 100 000 increased during the period in Brazil and Peru, which in 2019 had the highest rates in South America (82 DALYs for Brazil and 70 DALYs for Peru). In 2019, Brazil had the highest cocaine use disorder DALYs per 100 000 (45 DALYs), nearly double its rate in 1990. DALY rates were higher in males than females for each substance use disorder, except in Paraguay. The overall burden of substance use disorders was higher in males than in females, mainly because of cocaine use disorder and CAD, whereas for amphetamine use disorder, the difference between sexes was minimal, and for OUD there was no difference. For males and females, the highest rate of substance use disorders DALYs per 100 000 was for OUD except in Argentina (in males, 58 DALYs for cocaine use disorder *vs* 52 DALYs for OUD) and in Paraguay (in females, 77 for amphetamine use disorder *vs* 50 for OUD). CAD DALY rates were generally the lowest among the substance use disorders for males and females. Amphetamine use disorder YLD rates were reasonably stable throughout the period and were highest in Peru, Paraguay, and Uruguay (>40 YLD per 100 000). For CAD, YLD rates were stable in all countries except Chile and Colombia. Cocaine use disorder YLD rates per 100 000 for the top four countries (Argentina, Uruguay, Chile, and Brazil) increased from 1990 to 2010 (eg, from 19 to 33 in Brazil), but decreased between 2010 and 2019 (eg, from 36 to 31 in Chile). For OUD, YLD rates showed a slight increase in most countries apart from Brazil, which increased from 52 in 1990 to 80 in 2019 and was top among the countries. Amphetamine use disorder YLL rates per 100 000 were highest in Suriname and Peru during the period, although in Suriname it increased from 2·7 in 2010 to 3·2 in 2019, whereas in Peru it decreased from 2·1 to 1·7. The highest YLL rate for cocaine use disorder was in Brazil, which increased from 3·7 in 1990 to 18·1 in 2019. Between 2000 and 2019, Chile and Uruguay showed the highest OUD YLL rates (11·6 for Chile and 10·9 for Uruguay). A high incidence of CAD was found in Chile, Colombia, Guyana, and Suriname. There were high incidences of amphetamine use disorder in Paraguay, cocaine use disorder in Argentina, and OUD in Ecuador. A decrease in annual prevalence for substance use disorders during the period was observed in Venezuela (amphetamine use disorder, CAD, and OUD), Brazil (CAD and amphetamine use disorder), Colombia (amphetamine use disorder and cocaine use disorder), Peru (amphetamine use disorder and cocaine use disorder), Chile and Suriname (amphetamine use disorder), Uruguay (CAD), and Bolivia (OUD). Overall, the cocaine use disorder burden stabilised then decreased. OUD was less prevalent than other substance use disorders but its burden was the highest.

**Interpretation:**

The decrease in the burden of cocaine use disorder probably reflects the success of national standardised treatment programmes. Programmes for amphetamine use disorder, CAD, and OUD management should be improved. We did not find an increase in CAD burden in Uruguay, the country with the highest degree of cannabis decriminalisation in the region. Countries in South America should improve monitoring of substance use disorders, including regular surveys to provide more accurate data on which to base policy decisions.

**Funding:**

The Bill & Melinda Gates Foundation.

## Introduction

Substance use disorders (not including alcohol or tobacco use disorders) account for 0·8% of all-cause disability-adjusted life-years (DALYs) globally.^1^ Opioid use disorder (OUD), amphetamine use disorder, cocaine use disorder, and cannabis use disorder (CAD) are the most prevalent recreational drug disorders worldwide.^1^ Despite not being the most prevalent substance use disorder, OUD is the largest contributor to the DALYs burden.^1^ Substance use disorder prevalences and burdens differ substantially between different regions of the world.^1^

South America shows a particular profile of substance use. The region is a major supplier of cocaine, has low rates of opioid use, contains the first country to legalise recreational cannabis, and has a high prevalence of crack and base paste (subtypes of cocaine) use. In a representative survey conducted in Brazil, lifetime usage prevalence was 0·3% for heroin and 2·9% for non-medical prescription opioids.^2^ Usage prevalence in Brazil was ten times lower for heroin^3^ and four times lower for opioids^4^ than in the USA during a similar period (ie, 2012–2015). In 2013, Uruguay became the first country to legalise recreational cannabis, thereby creating a regulatory model of production and supply for sales controlled by the government in registered drug stores.^5^ Many other countries in the region (ie, Chile, Peru, Colombia, Bolivia, and Argentina) have regulated cannabis production for scientific and medical purposes.^5^ Crack and base paste are commonly used in the region, especially among youth from economically disadvantaged backgrounds,^6^ and are associated with worse health outcomes than for traditional cocaine powder.^7^ South America is a major cocaine supplier, with at least 19 major illicit coca-growing regions,^7^ and includes the top three global producers of cocaine (ie, Colombia, Peru, and Bolivia). These three countries have an estimated combined production capacity of 2122 metric tons of cocaine per year, which represents most of the cocaine production capacity globally.^8^ Illegal drug cultivation has become part of the informal economy in the region.^8^ The region also contains large areas of cannabis cultivation.^9^

**Research in context**

**Evidence before this study**

South America has problems related to substance use; the region is the world's leading producer of cocaine and also produces a substantial amount of cannabis. Uruguay became the first country to legalise recreational cannabis in 2013. Crack and base paste are subtypes of cocaine that are commonly used in the region and have worse health outcomes than traditional cocaine powder. We searched PubMed on June 20, 2022, without any language restrictions, using the keywords [“burden” OR “DALYs”] AND [“cocaine” OR “opioids” OR “amphetamines” OR “cannabis”] AND [“South America” OR “Latin America”]. We found two studies that examined the differences between global regions in the burden related to opioids and cannabis, neither of which included data after 2010. In a second search of PubMed on June 20, 2022, we used the search terms [“burden” OR “DALYs”] AND [“cocaine” OR “opioids” OR “amphetamines” OR “cannabis”] AND [“Argentina” OR “Bolivia” OR “Brazil” OR “Chile” OR “Colombia” OR “Ecuador” OR “Guyana” OR “Paraguay” OR “Peru” OR “Suriname” OR “Uruguay” OR “Venezuela”]. We found 36 studies, none of which included comparisons between countries in the region. Some studies assessed the burden for one drug over time in individual countries (eg, cocaine in Brazil), or made comparisons with countries from other continents (eg, Europe *vs* Asia) for a short period. We did not find any studies reporting the burden of substance use disorders in the countries in South America.

**Added value of this study**

To our knowledge, this is the first study to investigate the burden of disease attributable to amphetamine, cannabis, cocaine, and opioid use disorders in South America. Together, these data enable the calculation of estimated years lived with disability, years of life lost, and disability-adjusted life-years for males and females in each of the region's countries, enabling an evaluation of the effects of public policies and changes in substance use behaviours over the past three decades.

**Implications of all the available evidence**

The longitudinal assessment of the burden of substance use disorders can inform public health policies. The burden of cocaine use disorder has fallen in some South American countries, but efforts to prevent and treat cocaine use disorders in the region must be maintained. The increased opioid use disorder burden in the region, plus the rising use of prescription opioids, suggest physician education for effective management and health surveillance services to monitor prescriptions in pharmacies are needed to prevent misuse. The burden of amphetamine use disorders has not changed, so management programmes are required, especially to prevent the non-medical use of stimulant drugs among young people.

South America's particular substance use profile, poverty and income inequality,^10^ and role as a supplier of cocaine make this region a unique place for substance use research. This study aims to provide a comprehensive description of the burden of substance use disorders in the region. We also calculated this burden for Latin America and the Caribbean, for comparison purposes.

## Methods

### Study design and data sources

The study investigated the burden of disease attributable to amphetamine use disorder, CAD, cocaine use disorder, and OUD in South America from 1990 to 2019, using the Global Burden of Diseases, Injuries, and Risk Factors Study (GBD) 2019 data analysed by sex and location. GBD data are not stratified by ethnicity or race. The unit of analysis was each country, in each year of the study period.

GBD 2019 estimated incidence, prevalence, mortality, years of life lost (YLL), years of life lived with disability (YLD), and DALYs caused by 369 diseases and injuries for two sexes (male and female) and 204 countries or territories.^11^ GBD 2019 complies with the Guidelines for Accurate and Transparent Health Estimates Reporting (GATHER) statement.^11^, ^12^, ^13^

The quality of the data has improved during the three decades of the GBD Study. Regular updates to GBD estimates, referred to as the GBD round, are a crucial milestone for ongoing estimation.^11^ For each GBD round, the entire time series back to 1990 is re-estimated using all available data and the best available methods to ensure the most complete and comparable set of estimates possible.^11^

The GBD data estimation process identifies multiple relevant data sources for each disease, including systematic reviews of published studies, government and international organisation websites, published reports, primary data sources (eg, the Demographic and Health Surveys Program), and contributions of datasets by GBD collaborators. Each newly identified and obtained data source is given a unique identifier by a librarian team and included in the Global Health Data Exchange (GHDx). The GHDx makes publicly available the metadata for each source included in the GBD and also the data, if the data provider permits doing so. Readers can use the GHDx source tool to identify which sources were used for estimating any disease or injury outcome in any given location.

All 12 South American countries were included in this study. We also calculated the substance use disorders burden for Latin America and the Caribbean. The estimation models (appendix pp 1–4) can be imputed from various data sources (depending on the country's resources for substance use disorders) obtained by direct methods (eg, surveys, surveillance, and vital statistics systems) and indirect ones (eg, treatment and services data, capture–recapture and back-projection estimates). Location-level covariates were included in the models for each substance use disorder (appendix pp 1–4). The appendix (pp 6–10) shows the data-quality rating per 5-year interval, the percentage well certified (ie, the percentage of fatalities allocated to specific GBD causes and the proportion of deaths not categorised as levels 1 or 2) across time series (appendix p 6), and the underlying indicators for percentage well certified for the data source, with the maximum percentage well certified in each 5-year time interval (appendix pp 7–10) for South American countries throughout the study. Argentina, Brazil, Chile, Colombia, Guyana, Uruguay, and Venezuela had high data-quality ratings. Bolivia had the lowest data-quality rating. Data quality improved over time in Brazil, Chile, Colombia, Guyana, Paraguay, Suriname, and Venezuela, and remained stable in Argentina, Bolivia, Ecuador, Peru, and Uruguay.

### Outcomes

Amphetamine use disorder, CAD, cocaine use disorder, and OUD included asymptomatic (ie, in people with substance use disorders who experienced no disability due to their disorder), mild, and severe dependence disorders for each substance. In a comparative risk assessment, GBD 2019 also quantified the burden due to amphetamine use disorder, CAD, cocaine use disorder, and OUD as risk factors for other health outcomes.^1^, ^14^ Literature reviews were used to estimate relative risks for drug use as a risk factor for different health effects (eg, infectious diseases, suicide, or mental disorders). The appendix (p 11) shows drug-dependence sequelae, health states, health-state lay descriptions, and global disability weightings. To measure population-attributable fractions, we used estimates of relative risk with Bayesian meta-regression model exposure prevalence. To calculate the attributable burden, these fractions were multiplied by related cause-specific DALYs.^1^, ^14^ The US National Epidemiological Survey on Alcohol and Related Conditions data were used as the gold-standard data to calculate the percentage of asymptomatic cases for each substance use disorder.^15^ Using these ratios, a mean disability weighting was calculated for each condition across the severity range, with a disability weighting of 0 for asymptomatic patients.^15^

GBD 2019 estimated each epidemiological quantity of interest—incidence, prevalence, mortality, YLD, YLL, and DALYs—for males, females, and for sexes combined. The GBD diseases and injuries analytical framework generated estimates for every year from 1990 to 2019.^11^ Diseases and injuries were organised into a hierarchy of causes, ranging from level 1 for the three broadest causes of death and disability, to level 4 for the most specific ones (level 1=non-communicable diseases; level 2=mental disorders; level 3=drug use disorders in general; level 4 includes amphetamine use disorder, CAD, cocaine use disorder, and OUD).^11^ The covariates used, their level, and the expected direction of covariates by sex used in the burden estimation models for amphetamine use disorder, cocaine use disorder, and OUD are shown in the appendix (pp 12–18).

### Statistical analysis

Analyses were completed with Python (version 3.6.2), Stata (version 13), and R (version 3.5.0). We used three main standardised tools (ie, Cause of Death Ensemble Model, spatio-temporal Gaussian process regression, and Bayesian meta-regression model) to model processed data to generate estimates of each quantity of interest by sex, location, and year (appendix pp 1–4).

Briefly, the cause of death ensemble model is a highly systematised tool to analyse cause of death data using an ensemble of different modelling methods for death rates or cause-specific mortality fractions with varying covariate choices that did best in out-of-sample predictive validity testing. The appendix (p 19) shows the Cause of Death Ensemble model predictive validity results by model type, sex, and age for amphetamine use disorder, cocaine use disorder, and OUD models. All of these results show good in-sample data (a proportion of the available information to predict values outside of the estimation period) and out-of-sample data (previously unobserved information from which it is possible to make only a prediction or forecast). Spatio-temporal Gaussian process regression is a set of regression methods that borrow strength between locations and over time for single metrics of interest, such as risk factor exposure or mortality rates. The meta-regression model is a Bayesian meta-regression tool that evaluates all available data on incidence, prevalence, remission, and mortality for a disease, enforcing consistency between epidemiological parameters.

Cause-specific death rates and cause-specific mortality fractions were calculated using the Cause of Death Ensemble model and spatio-temporal Gaussian process regression. Cause-specific deaths were adjusted to match the total all-cause deaths calculated as part of the GBD population, fertility, and mortality estimates. Deaths were multiplied by standard life expectancy at each age to calculate YLL. A Bayesian meta-regression modelling tool, DisMod-MR 2.1,^11^ was used to ensure consistency between incidence, prevalence, remission, excess mortality, and cause-specific mortality for most causes. Prevalence estimates were multiplied by the disability weighting for mutually exclusive sequelae of diseases and injuries to calculate the YLD. Uncertainty intervals (UIs) were generated for every metric using the 25th and 975th ordered 1000 draw values of the posterior distribution. All comparisons in this Article were based on point estimate comparisons, without considering the UIs because of the high expected heterogeneity.

### Role of the funding source

The funder of the study had no role in study design, data collection, data analysis, data interpretation, or writing of the report.

## Results

Our longitudinal sample included an average of 365·8 million (range 293·1–431·1 million) individuals; 50·7% (50·6–51·0) female, 49·3% (49·1–49·4) male, 38·6% (31·4–45·3) aged 0–19 years, 31·8% (31·3–32·2) aged 20–39 years, 19·7% (15·8–23·5) aged 40–59 years, 8·5% (6·7–11·4) aged 60–79 years, and 1·4% (0·7–2·1) aged 80 years or older (appendix p 23).

**Figure 1:**
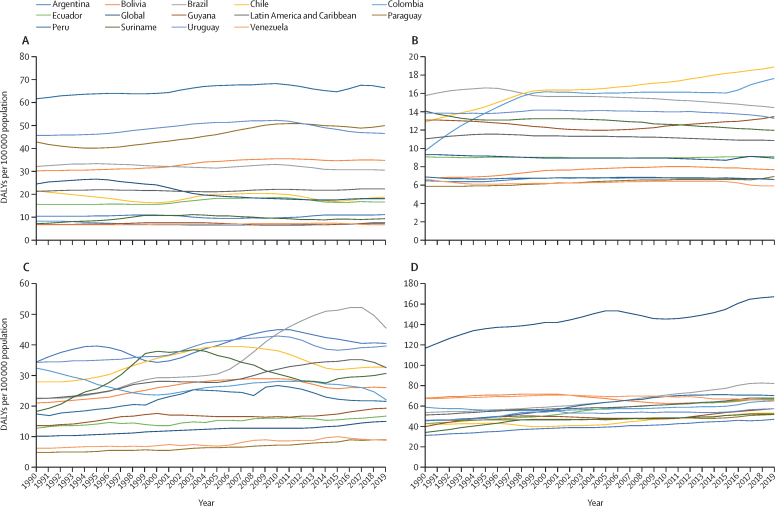
DALYs per 100 000 population by location, 1990–2019 Data are for all sexes and ages for amphetamine use disorder (A), cannabis use disorder (B), cocaine use disorder (C), and opioid use disorder (D). DALYs=disability-adjusted life-years.

Figure 1 shows all-age DALYs per 100 000 individuals by location from 1990 to 2019. The appendix (p 20) shows that all-age and age-standardised DALYs per 100 000 individuals by location for DALY rates for amphetamine use disorder remained stable between 1990 and 2019. In 2019, the highest amphetamine use disorder burdens per 100 000 population in South America were in Peru (66 DALYs), Paraguay (50 DALYs), and Uruguay (47 DALYs), all of which were higher than for Latin America and the Caribbean (22 DALYs) and global rates (18 DALYs). CAD DALY rates per 100 000 were also stable during this period, except in Chile and Colombia, which in 2019 had the highest DALYs (19 DALYs for Chile and 18 DALYs for Colombia), which were higher than Uruguay (13 DALYs), Latin America and the Caribbean (11 DALYs), and global rates (9 DALYs). In Brazil and Peru OUD DALYs per 100 000 increased during this period and, in 2019, these countries had the highest rates in South America (82 DALYs for Brazil and 70 DALYs for Peru).

During this period, there was more variation in DALY rates for cocaine use disorder than for OUD. In 2019, Brazil had the highest cocaine use disorder DALYs per 100 000 (45 DALYs) among countries in the region, nearly double its rate in 1990. By contrast, there were much smaller increases in the DALYs per 100 000 for Argentina, which had the second highest rate in 2019 (40 DALYs), and for Uruguay, which had the third highest rate that year (39 DALYs).

Apart from Paraguay, all the countries had higher DALYs rates in males than in females for each substance use disorder (appendix p 5). The overall burden of substance use disorders was higher in males than in females, mainly because of cocaine use disorder and CAD, whereas for amphetamine use disorder, the difference between sexes was minimal, and for OUD there was no difference. For males and females, the highest rate of substance use disorders was for OUD in all South American countries, except in Argentina (in males: 58 DALYs per 100 000 for cocaine use disorder *vs* 52 per 100 000 for OUD) and in Paraguay (in females: 77 DALYs per 100 000 for amphetamine use disorder *vs* 50 per 100 000 for OUD). Generally, cocaine use disorder had the second highest DALY rate among the substance use disorders for males and females. However, in Bolivia, Paraguay, Peru, and Uruguay, amphetamine use disorder DALYs were higher than cocaine use disorder DALYs for both males and females. CAD DALY rates were generally the lowest among the substance use disorders for males and females.

Table 1 shows all-age and age-standardised YLD rates per 100 000 individuals by location for 1990, 2000, 2010, and 2019, with sexes combined. The amphetamine use disorder YLD rates were reasonably stable throughout the period and were highest in Peru, Paraguay, and Uruguay (>40 YLD per 100 000 individuals). For CAD, YLD rates were stable in all the countries in the region, except Chile and Colombia where they increased to more than 15 YLD per 100 000. In South America as a whole, cocaine use disorder showed an irregular pattern between 1990 and 2019. Cocaine use disorder rates per 100 000 for the top four countries (Argentina, Uruguay, Chile, and Brazil) increased from 1990 to 2010 (eg, from 19 in 1990 to 33 in 2010, in Brazil), but then decreased between 1990 and 2019 (eg, from 36 in 2010 to 31 in 2019, in Chile). For OUD, YLD rates showed a slight increase in most countries, apart from in Brazil (in which there was a marked increase from 52 in 1990 to 80 in 2019, which was top among the South American countries).

**Table 1:** Years lived with disability per 100 000 people by location for sexes combined, 1990, 2000, 2010, and 2019

		**Years lived with disability per 100 000 people, all ages**				**Years lived with disability per 100 000 people, age standardised**			
		1990	2000	2010	2019	1990	2000	2010	2019
**Amphetamine use disorder**									
Global		17·84 (29·47–9·73)	15·72 (25·77– 8·67)	13·97 (22·79–7·69)	12·52 (20·78–6·82)	16·73 (27·56–9·31)	14·91 (24·40–8·24)	13·18 (21·57–7·24)	12·23 (20·25–6·67)
	Latin America and Caribbean	20·56 (34·45–10·96)	20·50 (33·95– 10·91)	21·19 (34·99–11·48)	21·06 (35·36–11·37)	19·18 (32·31–10·25)	18·91 (31·26–10·09)	19·42 (32·17–10·62)	19·83 (33·28–10·70)
	Argentina	10·26 (17·15–5·34)	10·69 (18·75– 5·82)	9·43 (15·23–5·26)	10·75 (18·22–5·58)	10·65 (17·79–5·54)	10·61 (18·43–5·76)	9·09 (14·67–5·08)	10·42 (17·74–5·43)
	Bolivia	29·36 (48·65–15·41)	30·50 (51·67– 15·77)	34·30 (57·21–18·26)	33·34 (56·30–17·99)	30·07 (49·86–15·87)	30·12 (50·41–15·78)	31·76 (52·96–17·04)	31·30 (52·43–17·09)
	Brazil	32·07 (53·61–16·94)	32·19 (53·80– 17·27)	32·75 (54·32–17·72)	30·16 (51·60–16·37)	29·29 (49·31–15·61)	28·93 (48·27–15·56)	29·17 (48·36–15·82)	28·55 (48·99–15·47)
	Chile	21·47 (36·86–11·03)	16·03 (26·34– 8·83)	19·55 (32·39–10·97)	17·92 (29·93–9·82)	18·94 (32·21–9·84)	15·23 (25·14–8·32)	18·53 (30·67–10·37)	17·35 (29·15–9·45)
	Colombia	6·64 (11·61–3·30)	6·21 (10·76– 3·09)	6·12 (10·36–3·11)	6·27 (10·79–3·19)	5·89 (10·11–3·04)	5·88 (10·12–2·99)	5·86 (9·90–3·02)	5·83 (10·07–2·98)
	Ecuador	14·92 (26·18–7·37)	15·04 (25·80– 7·61)	17·61 (29·77–8·88)	15·45 (26·63–7·91)	14·03 (24·30–7·15)	14·04 (23·92–7·16)	16·44 (27·53–8·39)	14·26 (24·48–7·36)
	Guyana	6·75 (12·15–3·22)	7·00 (12·54– 3·41)	5·95 (10·30–3·09)	6·86 (11·74–3·43)	5·94 (10·56–2·96)	6·43 (11·41–3·23)	5·93 (10·29–3·09)	5·97 (10·15–3·05)
	Paraguay	42·61 (71·01–21·97)	42·51 (71·49– 22·59)	50·31 (83·51–26·21)	49·31 (82·71–26·15)	42·92 (71·89–22·70)	42·62 (71·39–22·67)	45·30 (74·22–23·95)	43·75 (73·16–23·39)
	Peru	60·60 (101·14–31·39)	62·56 (104·06– 33·47)	66·05 (109·12–35·02)	64·75 (108·17–35·23)	58·37 (97·92–30·69)	58·31 (96·87–31·28)	60·86 (99·81–32·60)	60·88 (101·54–33·28)
	Suriname	6·74 (11·39–3·28)	7·05 (12·08– 3·60)	6·71 (11·36–3·36)	6·25 (10·56–3·27)	6·01 (10·01–2·99)	6·45 (11·02–3·28)	6·32 (10·68–3·18)	6·29 (10·68–3·23)
	Uruguay	45·57 (76·01–24·32)	48·37 (80·43– 26·87)	51·68 (86·13–28·01)	45·76 (77·12–25·56)	47·45 (79·01–25·26)	49·81 (83·39–27·71)	53·68 (89·63–28·94)	47·69 (80·94–26·21)
	Venezuela	6·34 (11·24–3·14)	6·32 (11·01– 3·21)	6·48 (11·09–3·22)	5·75 (9·67–2·93)	5·82 (10·16–2·95)	5·81 (10·00–3·00)	5·82 (9·89–2·97)	5·82 (9·85–2·95)
**Cannabis use disorder**									
Global		9·31 (14·59–5·56)	8·95 (13·97– 5·41)	8·91 (13·85–5·39)	8·92 (13·92–5·44)	8·78 (13·67–5·29)	8·50 (13·26–5·17)	8·42 (13·12–5·09)	8·79 (13·68–5·32)
	Latin America and Caribbean	11·03 (17·94–6·35)	11·34 (17·66– 6·71)	11·17 (17·42–6·66)	10·85 (16·66–6·49)	10·26 (16·47–6·04)	10·39 (15·97–6·20)	10·29 (15·96–6·18)	10·34 (15·91–6·19)
	Argentina	6·45 (10·11–3·71)	6·72 (10·09– 4·16)	6·76 (9·99–4·23)	6·59 (9·75–4·13)	6·52 (10·15–3·73)	6·49 (9·71–4·00)	6·53 (9·60–4·08)	6·58 (9·74–4·14)
	Bolivia	6·85 (10·63–3·89)	7·57 (11·70– 4·52)	7·97 (12·46–4·55)	7·66 (11·92–4·55)	6·97 (10·83–4·02)	7·31 (11·19–4·35)	7·35 (11·33–4·26)	7·38 (11·49–4·43)
	Brazil	15·74 (25·86–8·96)	15·64 (24·69–9·11)	15·31 (24·14–9·01)	14·44 (22·39–8·55)	14·46 (23·43–8·39)	13·96 (21·78–8·20)	13·94 (21·89–8·24)	13·88 (21·69–8·18)
	Chile	12·85 (19·27–7·92)	16·32 (23·87–10·51)	17·18 (25·08–11·10)	18·84 (27·54–12·21)	11·39 (16·98–7·07)	15·63 (22·90–10·05)	16·35 (23·91–10·57)	19·68 (28·75–12·75)
	Colombia	9·73 (15·18–5·73)	16·17 (24·13– 9·93)	16·11 (24·19–10·03)	17·58 (26·39–11·12)	8·81 (13·52–5·21)	14·91 (22·28–9·22)	15·05 (22·64–9·37)	16·76 (25·11–10·57)
	Ecuador	9·05 (14·54–4·96)	9·00 (14·05– 5·21)	8·99 (14·22–5·08)	9·09 (14·54–5·17)	8·54 (13·59–4·78)	8·42 (13·05–4·89)	8·46 (13·29–4·89)	8·48 (13·55–4·88)
	Guyana	13·08 (21·19–7·08)	12·27 (20·02– 6·93)	12·43 (19·91–7·11)	13·47 (23·57–6·95)	11·39 (18·18–6·30)	11·44 (18·58–6·47)	11·45 (18·40–6·53)	11·88 (20·61–6·26)
	Paraguay	5·86 (9·21–3·32)	6·13 (9·47– 3·42)	6·55 (10·29–3·73)	6·94 (11·23–3·93)	5·96 (9·10–3·42)	5·96 (9·18–3·34)	5·96 (9·29–3·42)	6·33 (10·10–3·65)
	Peru	6·88 (11·30–3·73)	6·75 (10·69– 3·79)	6·79 (10·67–3·84)	6·62 (10·45–3·69)	6·54 (10·66–3·64)	6·29 (9·85–3·57)	6·29 (9·86–3·58)	6·31 (9·97–3·53)
	Suriname	14·06 (24·92–7·23)	13·22 (21·98– 7·44)	12·63 (21·11–7·07)	11·96 (19·80–6·74)	12·54 (21·76–6·62)	11·98 (19·78–6·82)	11·94 (19·89–6·66)	11·89 (19·90–6·69)
	Uruguay	13·81 (21·77–7·88)	14·13 (21·00– 8·56)	13·98 (20·84–8·63)	13·24 (20·03–8·00)	14·27 (22·50–8·15)	14·55 (21·60–8·84)	14·57 (21·71–9·01)	14·25 (21·66–8·57)
	Venezuela	6·64 (11·01–3·69)	6·18 (10·07– 3·46)	6·42 (10·15–3·60)	5·91 (9·26–3·43)	6·32 (10·28–3·59)	5·83 (9·34–3·32)	5·87 (9·30–3·30)	5·80 (9·17–3·33)
**Cocaine use disorder**									
Global		7·86 (11·69–4·75)	7·80 (11·45– 4·75)	7·96 (11·62–4·91)	7·23 (10·67–4·40)	7·77 (11·55–4·7)4	7·62 (11·17–4·66)	7·60 (11·08–4·69)	7·02 (10·34–4·28)
	Latin America and Caribbean	16·24 (25·14–9·17)	19·04 (29·29– 10·99)	22·47 (34·17–13·27)	19·28 (29·48–11·17)	15·80 (24·13–9·19)	18·05 (27·52–10·57)	20·90 (31·76–12·39)	18·34 (28·08–10·62)
	Argentina	34·04 (52·38–19·48)	33·69 (49·93– 20·28)	44·25 (63·14–28·92)	39·68 (60·07–23·47)	34·86 (53·63–19·91)	33·24 (49·11–20·03)	42·75 (60·97–27·95)	38·39 (58·46–22·66)
	Bolivia	14·49 (23·86–7·94)	17·63 (28·29– 9·90)	20·09 (32·31–11·48)	16·48 (26·07–8·94)	14·84 (23·79–8·33)	17·17 (27·03–9·92)	18·54 (29·27–10·71)	16·05 (25·10–8·71)
	Brazil	18·59 (28·99–10·72)	24·72 (38·37– 14·25)	32·83 (49·75–19·37)	27·29 (41·22–15·95)	18·09 (27·82–10·72)	22·74 (34·62–13·24)	30·03 (45·47–17·74)	25·82 (39·36–15·10)
	Chile	27·52 (42·46–15·64)	34·88 (51·22– 22·43)	36·48 (53·72–22·41)	30·80 (47·14–17·36)	25·55 (39·11–14·79)	33·28 (48·73–21·31)	34·05 (50·31–20·96)	30·02 (47·05–16·77)
	Colombia	19·81 (31·17–10·74)	20·10 (31·76– 11·11)	24·26 (36·19–14·81)	16·51 (26·21–9·23)	18·35 (28·45–10·16)	18·98 (29·62–10·74)	22·86 (34·14–13·92)	15·57 (24·78–8·67)
	Ecuador	7·97 (13·23–4·20)	8·89 (14·59– 4·70)	9·43 (15·30–5·16)	8·95 (14·42–4·81)	7·66 (12·31–4·18)	8·40 (13·62–4·48)	8·94 (14·26–4·97)	8·41 (13·49–4·57)
	Guyana	13·33 (21·59–7·21)	13·03 (21·47– 6·84)	13·29 (21·50–7·28)	14·01 (22·22–7·63)	12·16 (19·11–6·82)	12·50 (20·10–6·68)	12·64 (20·47–7·04)	12·61 (19·90–6·96)
	Paraguay	4·05 (6·75–2·09)	4·48 (7·48– 2·34)	4·87 (8·08–2·49)	4·74 (7·58–2·44)	4·20 (6·86–2·25)	4·43 (7·31–2·31)	4·48 (7·30–2·35)	4·37 (6·92–2·29)
	Peru	9·31 (15·03–4·97)	9·89 (16·32– 5·30)	10·36 (17·01–5·75)	9·06 (14·08–4·92)	8·93 (14·12–4·88)	9·21 (15·07–5·07)	9·60 (15·59–5·37)	8·70 (13·53–4·73)
	Suriname	15·40 (24·53–8·41)	18·55 (29·41– 10·27)	17·33 (27·70–9·77)	16·02 (25·39–8·99)	14·25 (22·19–7·93)	17·11 (26·89–9·54)	16·51 (26·33–9·33)	15·87 (25·30–8·84)
	Uruguay	33·80 (53·37–19·26)	34·84 (52·96– 20·20)	40·73 (60·84–24·45)	36·66 (56·59–20·83)	34·47 (54·66–19·60)	35·22 (53·74–20·25)	41·19 (61·47–24·63)	37·28 (58·04–20·95)
	Venezuela	3·52 (5·94–1·78)	3·73 (6·08– 1·94)	3·99 (6·48–2·02)	3·57 (5·75–1·95)	3·34 (5·42–1·76)	3·48 (5·57–1·88)	3·64 (5·92–1·87)	3·54 (5·71–1·91)
**Opioid use disorder**									
Global		83·94 (119·22–56·27)	95·55 (133·05– 64·73)	103·19 (142·89–69·74)	114·16 (156·38–77·44)	84·16 (117·92–57·10)	93·65 (128·97–63·49)	98·95 (136·27–66·79)	109·86 (150·69–74·49)
	Latin America and Caribbean	48·19 (69·47–31·37)	52·67 (75·02– 34·51)	57·90 (81·89–38·01)	64·12 (88·96–42·98)	51·50 (72·12–33·93)	53·78 (74·94–35·70)	56·04 (78·27–37·21)	60·33 (83·70–40·46)
	Argentina	29·38 (41·69–19·39)	34·65 (48·70– 22·71)	37·79 (53·06–24·55)	42·47 (59·61–28·00)	30·54 (43·21–20·17)	34·92 (49·29–22·85)	36·42 (51·15–23·62)	39·83 (55·97–26·11)
	Bolivia	64·80 (94·55–41·26)	68·02 (99·20– 44·21)	58·83 (85·26–37·38)	61·85 (88·01–39·97)	77·42 (110·72–50·68)	78·14 (110·64–52·11)	62·78 (88·46–41·25)	64·18 (89·86–41·44)
	Brazil	52·37 (75·16–33·82)	57·22 (81·51– 36·94)	68·72 (98·11–45·16)	79·82 (110·54–53·23)	54·08 (75·85–35·30)	56·34 (79·10–37·08)	63·44 (89·65–41·83)	73·23 (101·49–48·81)
	Chile	39·34 (54·98–25·98)	36·01 (51·55– 23·31)	36·98 (53·69–23·44)	41·62 (58·64–26·79)	38·26 (52·70–25·44)	34·68 (49·50–22·50)	34·15 (49·91–21·51)	37·90 (54·30–24·08)
	Colombia	54·39 (80·09–34·51)	52·70 (76·70– 33·48)	55·68 (81·26–35·02)	62·17 (89·32–39·86)	55·78 (80·32–36·31)	53·90 (77·54–34·46)	54·91 (79·63–34·48)	57·73 (82·87–37·03)
	Ecuador	39·82 (59·18–25·06)	47·24 (68·38– 30·40)	57·97 (83·12–37·70)	63·15 (89·29–41·42)	45·31 (65·04–29·06)	50·82 (71·63–33·75)	60·08 (84·99–39·76)	62·63 (87·32–41·27)
	Guyana	37·65 (53·61–23·88)	46·96 (65·44– 30·97)	44·62 (61·70–29·87)	52·74 (73·39–36·00)	40·09 (54·88–26·04)	48·08 (66·32–32·18)	45·73 (63·07–30·86)	50·05 (68·60–34·26)
	Paraguay	44·68 (66·44–28·09)	45·32 (66·22– 28·10)	46·14 (67·48–29·05)	48·47 (70·57–30·85)	51·52 (73·40–33·06)	50·64 (72·41–32·07)	46·66 (66·85–29·57)	46·16 (66·32–29·59)
	Peru	42·20 (60·00–26·83)	50·47 (72·08– 33·58)	64·13 (90·07–42·69)	65·87 (92·20–43·58)	46·64 (65·20–30·26)	52·56 (73·37–34·51)	62·97 (87·67–42·29)	63·30 (88·40–42·14)
	Suriname	32·52 (47·32–20·87)	39·61 (55·73– 26·11)	44·18 (62·26–28·83)	47·62 (66·76–31·12)	33·62 (47·83–21·79)	38·76 (54·02–25·65)	42·25 (59·26–27·62)	46·24 (64·99–30·15)
	Uruguay	43·43 (63·85–27·31)	46·99 (68·27– 30·48)	44·86 (63·23–28·67)	46·63 (65·73–30·27)	43·44 (64·30–27·12)	46·31 (68·09–29·75)	43·20 (61·69–26·65)	44·13 (63·36–28·36)
	Venezuela	66·04 (99·12–40·50)	68·81 (99·88– 43·52)	66·20 (95·25–42·76)	62·16 (87·23–41·06)	70·21 (103·05–44·38)	69·77 (100·04–44·28)	63·25 (90·89–41·00)	60·21 (85·01–39·80)

Date are n (95% uncertainty interval).

**Figure 2:**
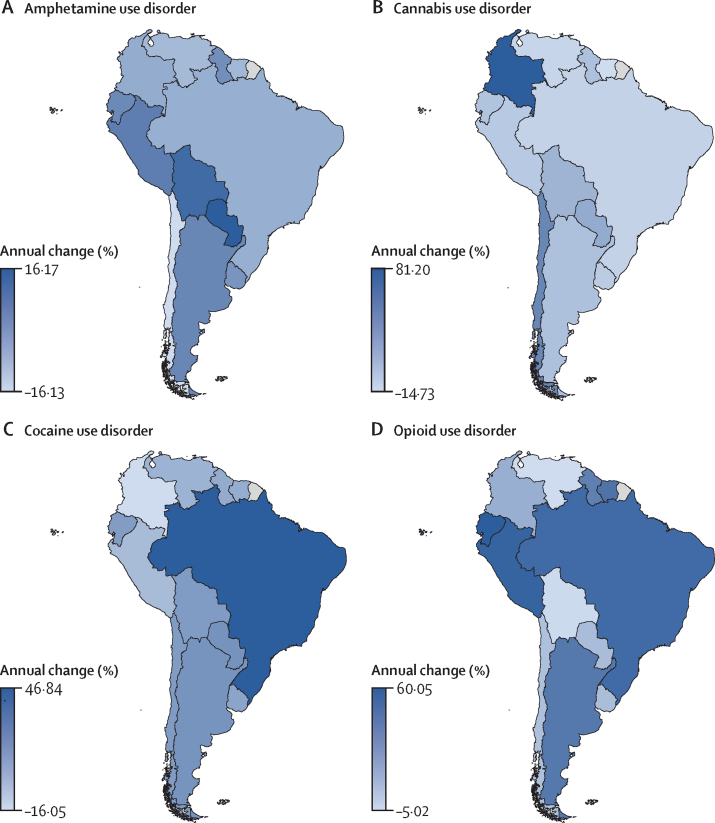
Annual percentage change in substance use disorders per 100 000 population by location, 1990–2019 Data are for all sexes and ages for amphetamine use disorder (A), cannabis use disorder (B), cocaine use disorder (C), and opioid use disorder (D).

Table 2 shows all-age and age-standardised YLL rates per 100 000 individuals by location for 1990, 2000, 2010, and 2019, with sexes combined. Suriname and Peru showed the highest amphetamine use disorder YLL rates per 100 000 population but differed in their trends; the rate in Suriname increased from 2·7 in 2010 to 3·2 in 2019, whereas in Peru it decreased from 2·1 to 1·7. The highest YLL rate for cocaine use disorder was in Brazil, in which the rate increased substantially from 3·7 in 1990 to 18·1 in 2019; Suriname and Peru also showed high YLL rates in 2019 (14·6 in Suriname and 12·3 in Peru). Between 1999 and 2019, Chile and Uruguay showed higher YLL rates for OUD than other countries in the region (eg, 11·60 for Chile and 10·9 for Uruguay *vs* 2·5–4·7 for other countries in 2019). Figure 2 shows the annual percentage change in substance use disorders per 100 000 population by location for 1990–2019. The appendix (pp 21–22) shows all-age and age-standardised prevalence and incidence rates per 100 000 individuals by location for 1990, 2010, and 2019. Uruguay (amphetamine use disorder, CAD, and cocaine use disorder), Brazil (CAD and OUD), Bolivia (amphetamine use disorder and OUD), and Peru (amphetamine use disorder and OUD) were among the top countries for the incidence of at least two substance use disorders during the period of the study. A high incidence of CAD was found in Chile, Colombia, Guyana, and Suriname. There were high incidences of amphetamine use disorder in Paraguay, cocaine use disorder in Argentina, and OUD in Ecuador. Some countries showed a decrease in annual prevalence during the 1990–2019 period for substance use disorders, as follows: Venezuela (amphetamine use disorder, CAD, and OUD), Brazil (CAD and amphetamine use disorder), Colombia (amphetamine use disorder and cocaine use disorder), Peru (amphetamine use disorder and cocaine use disorder), Chile and Suriname (amphetamine use disorder), Uruguay (CAD), and Bolivia (OUD).

**Table 2:** Years of life lost per 100 000 people by location for sexes combined, 1990, 2000, 2010, and 2019

		**Years of life lost per 100 000 people, all ages**				**Years of life lost per 100 000 people, age standardised**			
		1990	2000	2010	2019	1990	2000	2010	2019
**Amphetamine use disorder**									
Global		6·67 (9·75–5·64)	8·31 (10·09–7·54)	4·07 (4·28–3·90)	5·56 (6·04–5·13)	6·53 (9·53–5·55)	8·08 (9·80–7·33)	3·89 (4·09–3·73)	5·31 (5·78–4·89)
	Latin America and Caribbean	0·70 (0·74–0·66)	1·06 (1·12–1·00)	0·91 (0·96–0·87)	1·21 (1·41–1·03)	0·77 (0·81–0·73)	1·08 (1·14–1·03)	0·88 (0·93–0·84)	1·14 (1·32–0·97)
	Argentina	0·14 (0·17–0·12)	0·23 (0·27–0·20)	0·23 (0·26–0·20)	0·33 (0·38–0·28)	0·15 (0·17–0·13)	0·23 (0·27–0·20)	0·22 (0·25–0·19)	0·31 (0·36–0·27)
	Bolivia	0·85 (1·30–0·59)	1·10 (1·59–0·77)	1·21 (1·79–0·81)	1·41 (2·12–0·88)	1·05 (1·56–0·74)	1·29 (1·82–0·91)	1·32 (1·90–0·89)	1·52 (2·29–0·96)
	Brazil	0·11 (0·12–0·11)	0·12 (0·13–0·12)	0·24 (0·25–0·23)	0·40 (0·44–0·35)	0·12 (0·13–0·11)	0·12 (0·13–0·12)	0·22 (0·24–0·21)	0·37 (0·41–0·32)
	Chile	0·16 (0·19–0·14)	0·23 (0·26–0·20)	0·71 (0·80–0·63)	0·89 (1·03–0·75)	0·16 (0·19–0·14)	0·22 (0·25–0·19)	0·65 (0·73–0·57)	0·80 (0·93–0·67)
	Colombia	1·71 (1·95–1·51)	0·51 (0·58–0·46)	0·56 (0·64–0·50)	0·84 (1·17–0·52)	1·81 (2·02–1·61)	0·53 (0·59–0·47)	0·55 (0·63–0·49)	0·78 (1·09–0·48)
	Ecuador	0·60 (0·72–0·49)	0·58 (0·67–0·51)	0·90 (1·06–0·77)	1·15 (1·52–0·84)	0·71 (0·85–0·58)	0·64 (0·74–0·56)	0·94 (1·11–0·80)	1·15 (1·52–0·85)
	Guyana	0·07 (0·09–0·06)	0·63 (0·77–0·51)	0·47 (0·57–0·38)	0·76 (1·06–0·54)	0·08 (0·09–0·06)	0·64 (0·77–0·52)	0·46 (0·57–0·38)	0·74 (1·03–0·52)
	Paraguay	0·11 (0·16–0·08)	0·17 (0·22–0·14)	0·35 (0·44–0·29)	0·62 (0·85–0·43)	0·13 (0·18–0·09)	0·19 (0·23–0·16)	0·35 (0·43–0·29)	0·59 (0·80–0·41)
	Peru	1·07 (1·36–0·81)	1·52 (1·91–1·20)	2·15 (2·72–1·68)	1·72 (2·42–1·20)	1·18 (1·51–0·89)	1·57 (1·97–1·25)	2·12 (2·68–1·66)	1·66 (2·34–1·16)
	Suriname	0·47 (0·60–0·34)	3·74 (4·67–3·00)	2·74 (3·36–2·22)	3·16 (4·11–2·39)	0·52 (0·66–0·38)	3·67 (4·55–2·97)	2·57 (3·11–2·10)	3·01 (3·92–2·28)
	Uruguay	0·17 (0·19–0·15)	0·48 (0·53–0·42)	0·57 (0·64–0·50)	0·80 (0·92–0·70)	0·17 (0·19–0·15)	0·47 (0·52–0·41)	0·54 (0·61–0·48)	0·75 (0·87–0·65)
Venezuela		0·29 (0·38–0·24)	0·45 (0·52–0·39)	0·69 (0·78–0·61)	0·87 (1·17–0·63)	0·34 (0·42–0·28)	0·47 (0·54–0·41)	0·67 (0·76–0·59)	0·82 (1·11–0·59)
**Cannabis use disorder**									
Global		9·31 (14·59–5·56)	8·95 (13·97– 5·41)	8·91 (13·85–5·39)	8·92 (13·92–5·44)	8·78 (13·67–5·29)	8·50 (13·26–5·17)	8·42 (13·12–5·09)	8·79 (13·68–5·32)
Latin America and Caribbean		11·03 (17·94–6·35)	11·34 (17·66– 6·71)	11·17 (17·42–6·66)	10·85 (16·66–6·49)	10·26 (16·47–6·04)	10·39 (15·97–6·20)	10·29 (15·96–6·18)	10·34 (15·91–6·19)
Argentina		6·45 (10·11–3·71)	6·72 (10·09– 4·16)	6·76 (9·99–4·23)	6·59 (9·75–4·13)	6·52 (10·15–3·73)	6·49 (9·71–4·00)	6·53 (9·60–4·08)	6·58 (9·74–4·14)
Bolivia		6·85 (10·63–3·89)	7·57 (11·70– 4·52)	7·97 (12·46–4·55)	7·66 (11·92–4·55)	6·97 (10·83–4·02)	7·31 (11·19–4·35)	7·35 (11·33–4·26)	7·38 (11·49–4·43)
Brazil		15·74 (25·86–8·96)	15·64 (24·69–9·11)	15·31 (24·14–9·01)	14·44 (22·39–8·55)	14·46 (23·43–8·39)	13·96 (21·78–8·20)	13·94 (21·89–8·24)	13·88 (21·69–8·18)
Chile		12·85 (19·27–7·92)	16·32 (23·87–10·51)	17·18 (25·08–11·10)	18·84 (27·54–12·21)	11·39 (16·98–7·07)	15·63 (22·90–10·05)	16·35 (23·91–10·57)	19·68 (28·75–12·75)
Colombia		9·73 (15·18–5·73)	16·17 (24·13– 9·93)	16·11 (24·19–10·03)	17·58 (26·39–11·12)	8·81 (13·52–5·21)	14·91 (22·28–9·22)	15·05 (22·64–9·37)	16·76 (25·11–10·57)
Ecuador		9·05 (14·54–4·96)	9·00 (14·05– 5·21)	8·99 (14·22–5·08)	9·09 (14·54–5·17)	8·54 (13·59–4·78)	8·42 (13·05–4·89)	8·46 (13·29–4·89)	8·48 (13·55–4·88)
Guyana		13·08 (21·19–7·08)	12·27 (20·02– 6·93)	12·43 (19·91–7·11)	13·47 (23·57–6·95)	11·39 (18·18–6·30)	11·44 (18·58–6·47)	11·45 (18·40–6·53)	11·88 (20·61–6·26)
Paraguay		5·86 (9·21–3·32)	6·13 (9·47– 3·42)	6·55 (10·29–3·73)	6·94 (11·23–3·93)	5·96 (9·10–3·42)	5·96 (9·18–3·34)	5·96 (9·29–3·42)	6·33 (10·10–3·65)
Peru		6·88 (11·30–3·73)	6·75 (10·69– 3·79)	6·79 (10·67–3·84)	6·62 (10·45–3·69)	6·54 (10·66–3·64)	6·29 (9·85–3·57)	6·29 (9·86–3·58)	6·31 (9·97–3·53)
Suriname		14·06 (24·92–7·23)	13·22 (21·98– 7·44)	12·63 (21·11–7·07)	11·96 (19·80–6·74)	12·54 (21·76–6·62)	11·98 (19·78–6·82)	11·94 (19·89–6·66)	11·89 (19·90–6·69)
Uruguay		13·81 (21·77–7·88)	14·13 (21·00– 8·56)	13·98 (20·84–8·63)	13·24 (20·03–8·00)	14·27 (22·50–8·15)	14·55 (21·60–8·84)	14·57 (21·71–9·01)	14·25 (21·66–8·57)
Venezuela		6·64 (11·01–3·69)	6·18 (10·07– 3·46)	6·42 (10·15–3·60)	5·91 (9·26–3·43)	6·32 (10·28–3·59)	5·83 (9·34–3·32)	5·87 (9·30–3·30)	5·80 (9·17–3·33)
**Cocaine use disorder**									
Global		2·20 (2·72–2·04)	3·92 (4·44–3·72)	4·67 (5·00–4·51)	7·68 (8·45–7·17)	2·27 (2·83–2·10)	3·89 (4·40–3·70)	4·49 (4·81–4·34)	7·28 (8·03–6·79)
	Latin America and Caribbean	6·27 (6·53–6·00)	8·98 (9·43–8·62)	9·44 (9·85–9·06)	13·15 (14·44–11·95)	6·50 (6·76–6·25)	8·88 (9·30–8·52)	8·96 (9·33–8·60)	12·41 (13·63–11·27)
	Argentina	0·32 (0·37–0·27)	0·50 (0·56–0·44)	0·57 (0·65–0·50)	0·80 (0·90–0·70)	0·33 (0·38–0·28)	0·50 (0·57–0·44)	0·56 (0·63–0·49)	0·74 (0·84–0·65)
	Bolivia	6·46 (8·89–4·81)	8·39 (11·23–6·14)	8·82 (12·47–6·14)	9·53 (13·34–6·29)	7·40 (9·89–5·65)	9·16 (11·94–6·93)	9·10 (12·59–6·47)	9·95 (13·89–6·69)
	Brazil	3·72 (3·94–3·51)	4·42 (4·67–4·20)	10·91 (11·54–10·33)	18·14 (19·90–16·67)	3·84 (4·06–3·65)	4·27 (4·49–4·07)	9·98 (10·54–9·44)	17·01 (18·71–15·63)
	Chile	0·30 (0·35–0·26)	0·76 (0·87–0·66)	1·45 (1·66–1·27)	1·83 (2·14–1·56)	0·31 (0·36–0·27)	0·73 (0·84–0·64)	1·32 (1·51–1·16)	1·63 (1·90–1·38)
	Colombia	12·53 (14·00–11·12)	3·50 (3·95–3·09)	3·66 (4·18–3·23)	5·46 (7·62–3·53)	12·33 (13·63–11·11)	3·45 (3·88–3·07)	3·53 (4·02–3·12)	5·11 (7·13–3·31)
	Ecuador	4·76 (5·57–4·06)	4·72 (5·36–4·17)	6·54 (7·62–5·63)	7·70 (10·16–5·88)	5·34 (6·12–4·64)	4·97 (5·57–4·43)	6·62 (7·65–5·72)	7·55 (9·90–5·75)
	Guyana	0·25 (0·32–0·19)	4·55 (5·57–3·75)	3·11 (3·74–2·54)	5·18 (7·11–3·69)	0·26 (0·33–0·20)	4·49 (5·50–3·72)	3·08 (3·71–2·51)	4·91 (6·71–3·50)
	Paraguay	0·78 (1·05–0·55)	1·04 (1·29–0·84)	2·23 (2·82–1·84)	4·23 (5·82–2·99)	0·85 (1·13–0·62)	1·10 (1·35–0·90)	2·16 (2·67–1·81)	3·98 (5·44–2·82)
	Peru	8·07 (10·05–6·31)	12·05 (14·94–9·71)	16·20 (20·02–12·84)	12·34 (17·10–8·77)	8·25 (10·20–6·51)	11·76 (14·51–9·55)	15·40 (19·13–12·27)	11·86 (16·43–8·46)
	Suriname	2·78 (3·40–1·99)	19·18 (23·08–15·85)	13·11 (15·59–10·99)	14·57 (18·60–11·13)	2·91 (3·55–2·17)	18·54 (22·24–15·40)	12·26 (14·59–10·32)	14·13 (17·99–10·80)
	Uruguay	0·44 (0·51–0·38)	1·21 (1·38–1·07)	2·02 (2·30–1·78)	2·83 (3·39–2·33)	0·45 (0·52–0·38)	1·21 (1·38–1·07)	1·99 (2·29–1·75)	2·79 (3·35–2·28)
	Venezuela	2·58 (3·15–2·16)	3·23 (3·67–2·85)	4·60 (5·16–4·06)	5·09 (6·82–3·65)	2·81 (3·33–2·40)	3·27 (3·67–2·92)	4·42 (4·94–3·93)	4·92 (6·62–3·52)
**Opioid use disorder**									
Global		32·65 (34·88–30·16)	46·26 (48·12–44·28)	42·22 (43·40–41·13)	52·91 (55·66–50·79)	34·06 (36·37–31·41)	46·01 (47·85–44·04)	40·64 (41·78–39·58)	50·51 (53·21–48·46)
	Latin America and Caribbean	3·00 (3·18–2·84)	4·22 (4·42–4·03)	3·37 (3·53–3·22)	3·96 (4·53–3·46)	3·37 (3·56–3·19)	4·37 (4·56–4·18)	3·33 (3·49–3·18)	3·74 (4·28–3·27)
	Argentina	1·82 (2·04–1·64)	3·22 (4·11–2·67)	4·07 (5·06–3·32)	4·32 (4·88–3·80)	1·89 (2·11–1·70)	3·23 (4·10–2·69)	4·06 (5·13–3·25)	4·05 (4·59–3·56)
	Bolivia	3·18 (4·14–2·39)	3·86 (5·01–2·93)	3·90 (5·49–2·70)	4·15 (5·98–2·71)	4·23 (5·51–3·17)	4·89 (6·26–3·76)	4·55 (6·26–3·23)	4·70 (6·73–3·15)
	Brazil	1·24 (1·32–1·17)	1·56 (1·65–1·48)	1·97 (2·07–1·88)	2·46 (2·71–2·23)	1·30 (1·38–1·23)	1·54 (1·62–1·47)	1·86 (1·95–1·77)	2·25 (2·47–2·04)
	Chile	3·08 (3·50–2·72)	3·72 (4·21–3·30)	10·41 (11·51–9·32)	11·60 (13·28–10·03)	2·98 (3·36–2·65)	3·55 (4·00–3·16)	9·56 (10·58–8·56)	10·67 (12·28–9·20)
	Colombia	4·47 (5·05–3·97)	2·63 (3·58–2·05)	2·75 (3·53–2·21)	2·78 (3·87–1·78)	4·97 (5·54–4·46)	2·60 (3·42–2·08)	2·72 (3·48–2·19)	2·60 (3·61–1·66)
	Ecuador	2·91 (3·48–2·44)	3·03 (3·45–2·65)	3·24 (3·86–2·73)	3·70 (4·94–2·72)	3·77 (4·45–3·20)	3·56 (4·02–3·14)	3·54 (4·17–3·01)	3·80 (5·08–2·81)
	Guyana	2·08 (2·53–1·66)	2·92 (3·54–2·39)	2·79 (3·29–2·34)	4·41 (5·91–3·23)	2·14 (2·59–1·73)	2·97 (3·57–2·46)	2·82 (3·31–2·37)	4·25 (5·72–3·13)
	Paraguay	1·02 (1·34–0·79)	1·67 (2·00–1·39)	2·31 (2·83–1·90)	3·02 (4·03–2·15)	1·19 (1·54–0·94)	1·85 (2·20–1·56)	2·43 (2·94–2·04)	2·98 (4·00–2·15)
	Peru	3·46 (4·70–2·59)	4·68 (5·96–3·63)	5·09 (6·47–4·00)	4·43 (6·28–3·00)	3·98 (5·20–3·05)	4·98 (6·19–3·90)	5·11 (6·45–4·04)	4·32 (6·10–2·94)
	Suriname	1·35 (1·77–0·98)	6·81 (8·60–5·32)	4·14 (5·22–3·33)	4·70 (6·61–3·37)	1·47 (1·92–1·09)	6·65 (8·38–5·26)	3·94 (4·95–3·18)	4·54 (6·39–3·25)
	Uruguay	2·56 (2·88–2·29)	7·10 (7·83–6·43)	9·28 (10·33–8·37)	10·92 (12·49–9·49)	2·56 (2·88–2·26)	6·88 (7·63–6·20)	8·80 (9·84–7·86)	10·14 (11·76–8·76)
	Venezuela	1·33 (1·62–1·09)	1·80 (2·04–1·60)	3·15 (4·29–2·51)	3·15 (4·24–2·24)	1·59 (1·89–1·32)	1·96 (2·18–1·76)	3·24 (4·38–2·58)	2·98 (4·00–2·13)

Date are n (95% uncertainty interval).

## Discussion

This study estimated incidence, prevalence, YLL, YLD, and DALYs attributed to amphetamine use disorder, CAD, cocaine use disorder, and OUD in South American countries from 1990 to 2019. Compared with global estimates using the same data source, countries in the region showed a higher burden of cocaine use disorder, similar amphetamine use disorder and CAD, and lower OUD. Despite the OUD burden being lower in South America than it is globally, it has slowly increased during the past three decades and has the heaviest burden among substance use disorders within the region. The cocaine use disorder burden grew more quickly than the OUD burden until 2010, at which time it plateaued, before decreasing in 2016–19, and it represents the second greatest burden among substance use disorders in South America. By contrast, the amphetamine use disorder and CAD burdens have been stable during the past three decades, with some country exceptions.

Despite a decrease in disability (DALYs) attributed to cocaine use disorder in 2016–19, the relevant burden in the region remains much higher than the global average. Cocaine use disorder burden is higher in both North and South America than globally.^1^ The burden for cocaine use was higher in South America, but lower globally, than for amphetamine use disorder or CAD.^1^, ^16^ South America is the top global cocaine supplier,^7^ and the high prevalence of cocaine use can be linked to the proximity of production and the low price of the drug, especially in the form of crack and base paste.^7^ Brazil, Argentina, and Uruguay contributed the most to the region's cocaine use disorder burden. Brazil and Uruguay were among the top global countries in DALYs attributable to the use of cocaine.^17^ In South America, Brazil had the highest rates for both YLD and YLL, with high amounts of both snorted and smoked cocaine reported in studies done in the past two decades,^18^ as one of the top global markets for buying cocaine.^19^ Cocaine use disorder affects individuals across several domains. A comparative study in Argentina found that people who smoke cocaine have broad vulnerability across different spheres (eg, work, education, housing, and social),^20^ which could be important factors for increased morbidity and mortality owing to cocaine use disorder in the region.^18^, ^20^ Base paste is a popular form of cocaine in South America.^21^, ^22^, ^23^ A retrospective naturalistic study^22^ (n =113) done in Uruguay showed that this type of cocaine was significantly associated with suicidal behaviours shortly after use. In Peru, which the current study found to have one of the top cocaine use disorder YLL within the region, base paste has almost the same prevalence of lifetime use (2·3%) as traditional cocaine powder (2·6%) among students aged 12–17 years.^21^

Since the start of the GBD, OUD burden estimates for South America have been lower than for global ones.^1^ Traditionally, heroin use has been low in South America,^24^ where the drug is expensive and inhaled or smoked rather than injected.^24^ Although OUD is less common than other substance use disorders are in South America, it creates the greatest burden. The OUD burden in South America (figure 1D) has increased continuously during the past 30 years. There is no obvious cause for this growth. Because individuals who use opioids often have conditions that are associated with increased risk of mortality and morbidity, the cause of death (eg, overdose *vs* pain-related clinical disease) or disability (eg OUD *vs* pain-related clinical disease) might be inaccurately reported. Therefore, the main comorbidities related to OUD could be responsible for the high associated burden and should be the focus of preventive interventions in the region.^25^ More accurate indicators for opioid use, prescribing practice, and usage hazards in Brazil are required, because current and acute (eg, pain-related) demands for improved opioid use and management appear to be increasing.^24^ Brazil and Peru have a considerable OUD burden, and the highest DALYs and YLD rates in South America. Surveys point to increases in sales of prescribed opioids^26^ and experimentation with heroin,^27^ and a longitudinal study^26^ (n=1 601 043) found a trend of increased sales of prescribed opioids, especially oxycodone, in Brazil from 2009 to 2015. In 2017, a national survey^21^ of Peruvian students aged 12–17 years found that 0·5% had already used heroin. Despite not showing high rates of OUD DALYs and YLD, Chile and Uruguay had the highest YLL for OUD since 2000. In an international longitudinal study^27^ that investigated drug overdose mortality from 2001 to 2015, Chile ranked among the top half of countries for average annual percentage of change in the rate of deaths from drug overdose, showing an increased trend in both males and females. Opioid misuse was classified as an emergent drug problem in Uruguay by the InterAmerican Commission for Drug Abuse Control and the Uruguayan government in 2020.^28^ In 2018, 4·9% of Uruguayans used prescription opioids.^28^

South American countries showed higher YLD and lower YLL compared with the global rates. Living with a disability has contributed more to the amphetamine use disorder burden rates than has mortality. YLL rates attributed to amphetamine use disorder in the region were much lower than global ones, even in the most affected countries (ie, Peru and Paraguay). Despite one school-based survey^29^ showing a lower lifetime-use prevalence for amphetamines than for cannabis and cocaine, Peru had a higher DALYs rate for amphetamine use disorder than for CAD^29^ and cocaine use disorder.^21^ Paraguayan females were found to be a risk group for amphetamine use disorder burden. Surveys in Paraguay found high prevalences of use of methylphenidate and non-prescribed stimulants among medical students^30^ and adolescent females.^31^

CAD burden has been stable throughout the past three decades in South America, except for Chile and Colombia, which have shown increased CAD burden during this period and reached twice the global CAD DALYs rate in 2019. These results are in line with recent studies. A cross-sectional study^32^ that compared university students from Bolivia, Ecuador, Peru, and Colombia found the highest prevalence in Columbia, with between two and six times the past-month cannabis use prevalence of the other countries. A longitudinal study^33^ showed a significant increase in the proportion of Chilean adolescents (in grades 8–12) who had used cannabis in the past year, from 2013 (13·6%) to 2017 (31·3%). Chile legalised medical use only of cannabis in 2015.^34^ Uruguay, which has legalised both recreational and medical use,^5^ showed a high, but stable, prevalence of cannabis use,^34^ and has shown a stable high CAD burden during the past three decades. Despite GBD not taking CAD into account for mortality, a cross-sectional study^35^ (n=1462) in Colombia suggested an association between cannabis consumption and suicide risk.

Most countries in South America showed increased YLD rates for amphetamine use disorder, CAD, and cocaine use disorder whereas, globally, these rates have decreased since 1990. By contrast, changes in South American and global YLL rates were similar. Health reforms during the structural adjustment programmes in South America in 1980–2000 reduced poverty and social inequity, and improved health-care access, leading to a reduction in mortality but an increase in YLD.^36^

Our findings can be considered in relation to the region's treatment access, decriminalisation, and stigma reduction programmes. Although Brazil has the highest rates of DALYs owing to OUD, it also has the highest extent of approval and availability of methadone and naloxone programmes in the region.^37^ The establishment of standardised nationwide treatment programmes appears to have decreased the cocaine use disorder burden, for example, in Argentina, Brazil, Colombia, and Peru.^37^ By contrast, the burden of OUD and amphetamine use disorder appears to be unaffected by these programmes. A high amount of stigma is attached to substance use disorders throughout Latin America,^38^ which is an important barrier to obtaining treatment for Latin Americans who live with a substance use disorder.^38^ We did not find an increase in the burden associated with CAD owing to the liberalisation of cannabis use in Uruguay, the country with the highest cannabis decriminalisation rates in the region.^39^ Colombia, which had one of the lowest rates of decriminalisation of cannabis use for non-medical purposes in the region,^40^ had a sizable increase in the CAD burden. Therefore, our data do not support a link between decriminalisation and the rise in the CAD burden.

The study had some limitations. First, we assumed that uncertainty (expressed in UIs) is independent between YLL and YLD but little empirical evidence exists to establish this correlation. However, this assumption could result in an underestimation of the overall uncertainty for DALYs. Second, the availability of data for YLL and YLD estimations influences DALY estimates. Recent changes in health states might not have been captured in our estimates, because of time lags in the reporting of health data by nations and the subsequent incorporation of these data into the GBD estimate. Wider intervals of uncertainty reflect the data scarcity for a specific location. Despite most South American countries showing high-quality data ratings in general, the most reliable data might come from mortality or vital statistics that do not usually involve drug testing for multiple substances and from national surveys that are conducted only once every 3–5 years. Third, although we included several sources of uncertainty in our estimates, we have not been able to integrate uncertainty into the covariates used for the cause of death and non-fatal models.

In conclusion, the burden of cocaine use disorder, a major problem in the region owing to the proximity of production sites, has fallen in some South American countries, possibly owing to national treatment programmes (eg, in Argentina, Brazil, Colombia, and Peru). The burden of amphetamine use disorder and of CAD has remained unchanged in most countries, so efforts should be made to improve management protocols for these disorders. Uruguay, which legalised cannabis use in the past decade, has shown a high but stable prevalence and burden of CAD over the past three decades. The increased OUD burden in the region, especially in Brazil, makes it important to improve the availability of methadone and naloxone. Finally, better substance use disorder monitoring in the region is required, with systematic surveys, to improve the quality of data and thus strengthen the evidence base for policy makers.

## Data sharing

All GBD data are publicly available (https://ghdx.healthdata.org/). The statistical code used for GBD estimation is publicly available online (http://ghdx.healthdata.org/gbd-2019/code).

For more on the **Demographic and Health Surveys Program** see https://dhsprogram.com/

For more on the **Global Health Data Exchange source tool** see https://ghdx.healthdata.org/

For more on the **National Epidemiological Survey on Alcohol and Related Conditions data** see https://catalog.data.gov/dataset/national-epidemiologic-survey-on-alcohol-and-related-conditions-nesarc-iii#sec-dates

## Declaration of interests

JMC-M reports grants from the French National Institute for Cancer, Pfizer, and Sanofi, and consulting fees from L'Oreal, all outside of the submitted work. ARB reports grants from the São Paulo Research State Foundation, outside of the submitted work. Y-PW reports support for attending meetings or travel from the Department of Psychiatry, Medical School, University of Sao Paulo and from Lundbeck Brasil, and payment or honoraria for lectures, presentations, speakers bureaus, manuscript writing, or educational events from Meda Pharma. All other authors declare no competing interests.
