# Supplementary material for: Burden of disease due to amphetamines, cannabis, cocaine, and opioid use disorders in South America, 1990–2019: a systematic analysis of the Global Burden of Disease Study 2019
Source: Lancet Psychiatry. 2023 Feb;10(2):85–97. doi: 10.1016/S2215-0366(22)00339-X (PMC9870787; doi:10.1016/S2215-0366(22)00339-X)
Supplement: Supplementary appendix [file mmc1.pdf]

THE LANCET
Psychiatry

Supplementary appendix

This appendix formed part of the original submission and has been peer reviewed. We post it as supplied by the authors.

Supplement to: Castaldelli-Maia JM, Wang Y-P, Brunoni AR, et al. Burden of disease due to amphetamines, cannabis, cocaine, and opioid use disorders in South America, 1990–2019: a systematic analysis of the Global Burden of Disease Study 2019. *Lancet Psychiatry* 2023; **10:** 85–95.

Appendix. Supplementary File for the Article "Burden of disease due to amphetamines, cannabis, cocaine, and opioid use disorders in South America, 1990–2019: a systematic analysis of the Global Burden of Disease Study 2019"

**Supplementary File**	**Page**
Figure S1. AmUD burden estimation model	1
Figure S2. CaUD burden estimation model	2
Figure S3. CoUD burden estimation model	3
Figure S4. OpUD burden estimation model	4
Figure S5 presents sex differences regarding DALYs by each SUD. Apart from Paraguay, all the countries had higher DALYs by SUDs (AmUD + CaUD + CoUD + OpUD) in males, compared to females	5
Table S1. Data quality rating from 0 to 5 stars, maximum percent well certified per 5-year interval and percent well certified across time series for South American countries, 1980-2019	6
Table S2. Underlying indicators for percent well-certified for data source with maximum percent well certified in each 5-year time interval for the South American countries, 1980-2019	7
Table S3. Drug dependence sequelae, health states, health state lay descriptions, and disability weights	11
Table S4. CODEm covariates used, level of covariate, and expected direction of covariate by cause, sex, and age	12
Table S5. CODEm predictive validity results by cause, model type, sex, and age	19
Table S6. All-age and age standardized DALYs rates per 100,000 individuals by location for 1990, 2010, and 2019, both sexes combined	20
Table S7. All-age and age standardized prevalence rates per 100,000 individuals by location for 1990, 2010, and 2019, both sexes combined	21
Table S8. All-age and age standardized incidence rates per 100,000 individuals by location for 1990, 2010, and 2019, both sexes combined	22
Table S9. Estimates of the South American Population, Global Burden of Disease Study, 1990-2019	23

Figure S1. AmUD burden estimation model

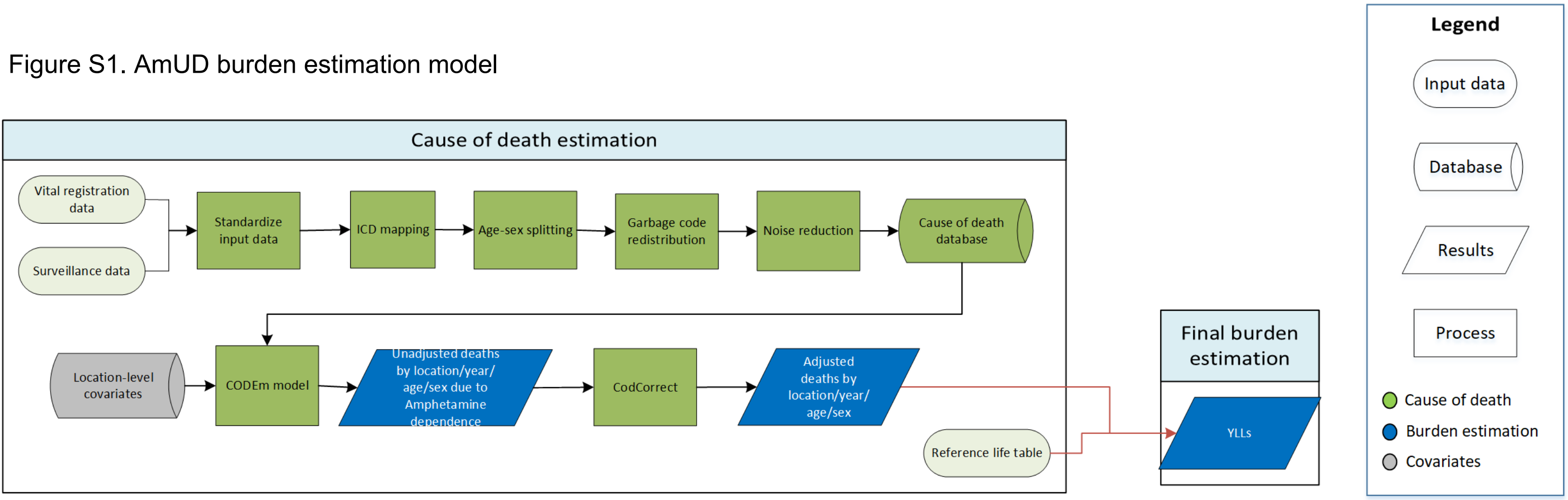

Figure S2. CaUD burden estimation model

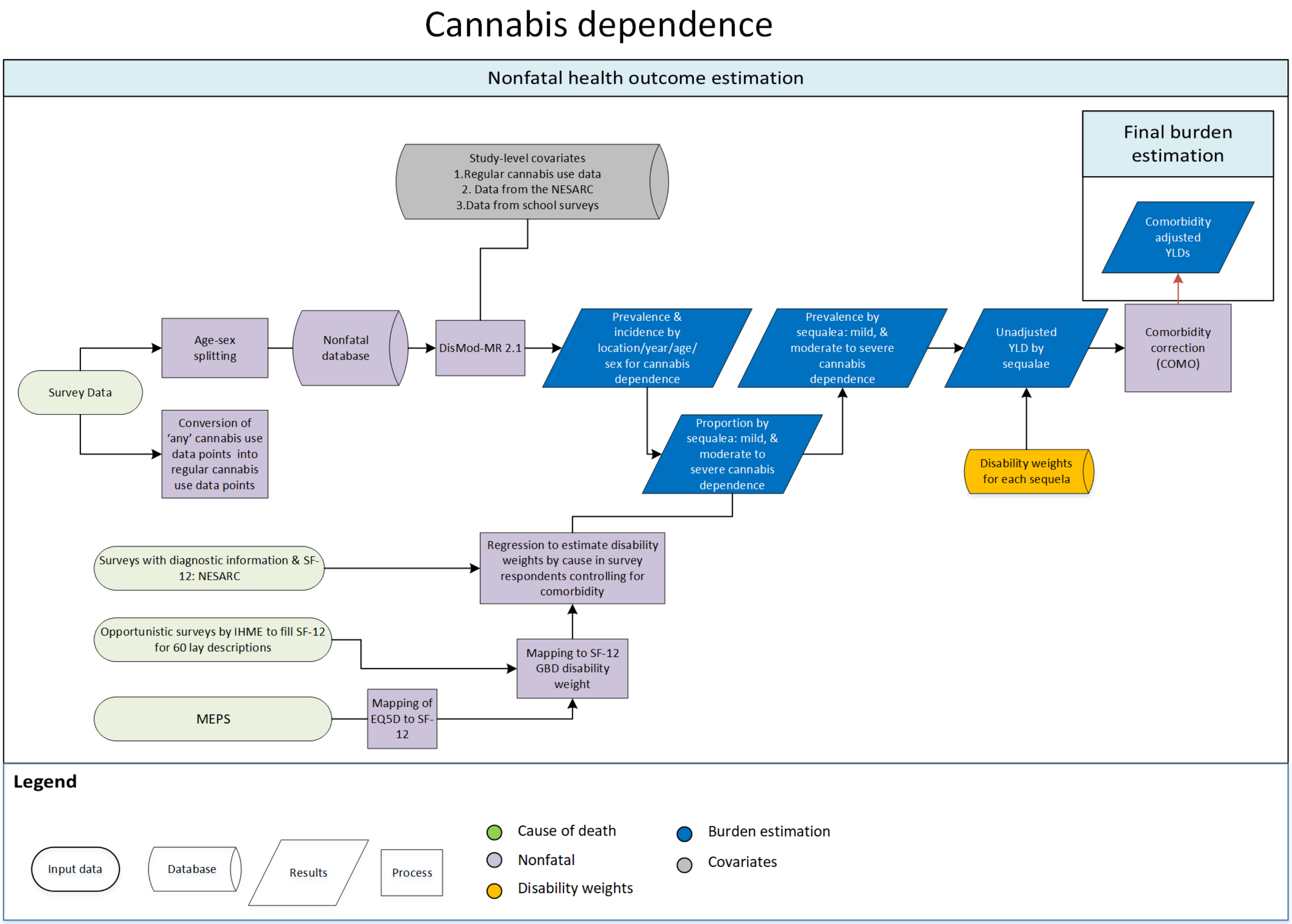

Figure S3. CoUD burden estimation model

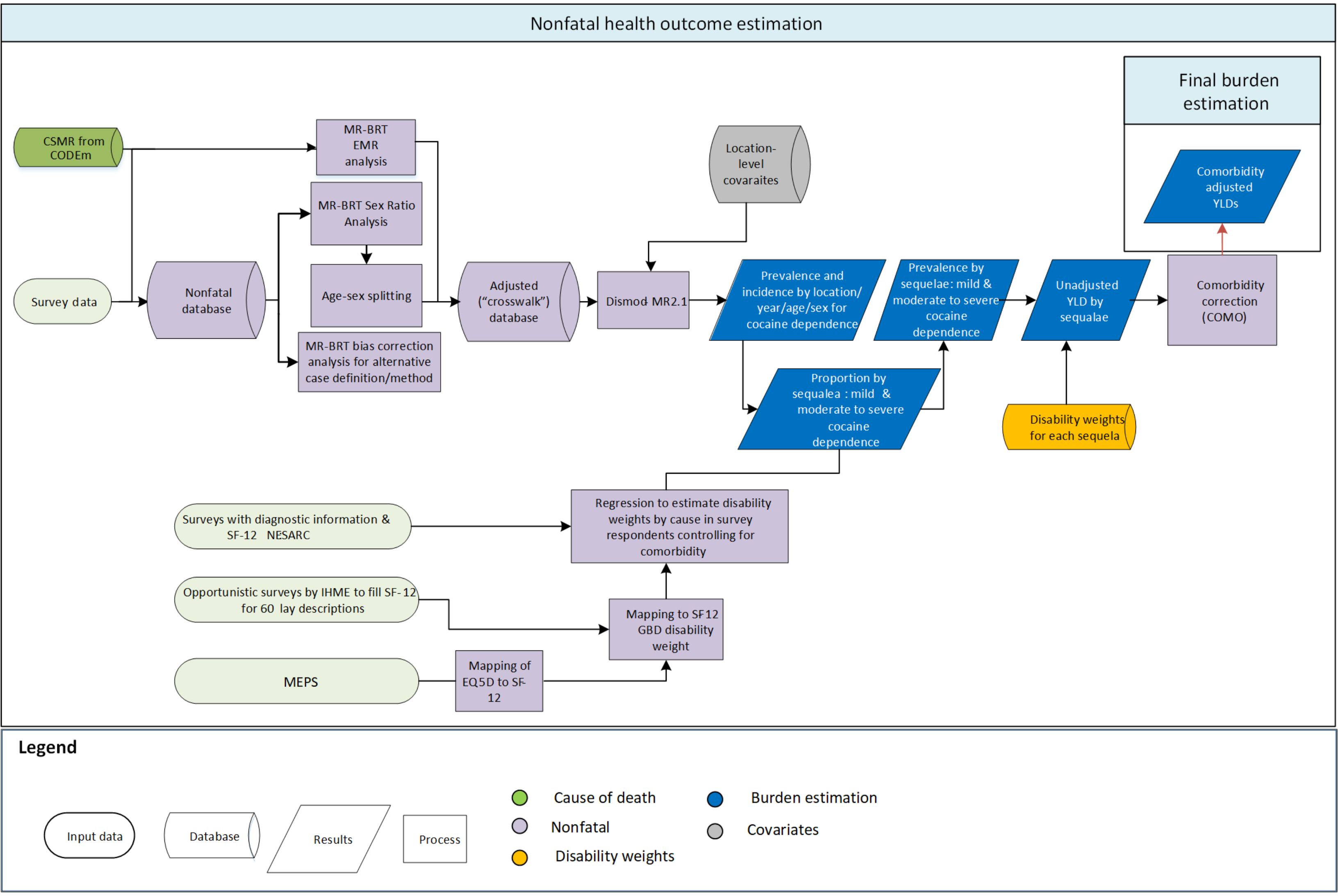

Figure S4. OpUD burden estimation model

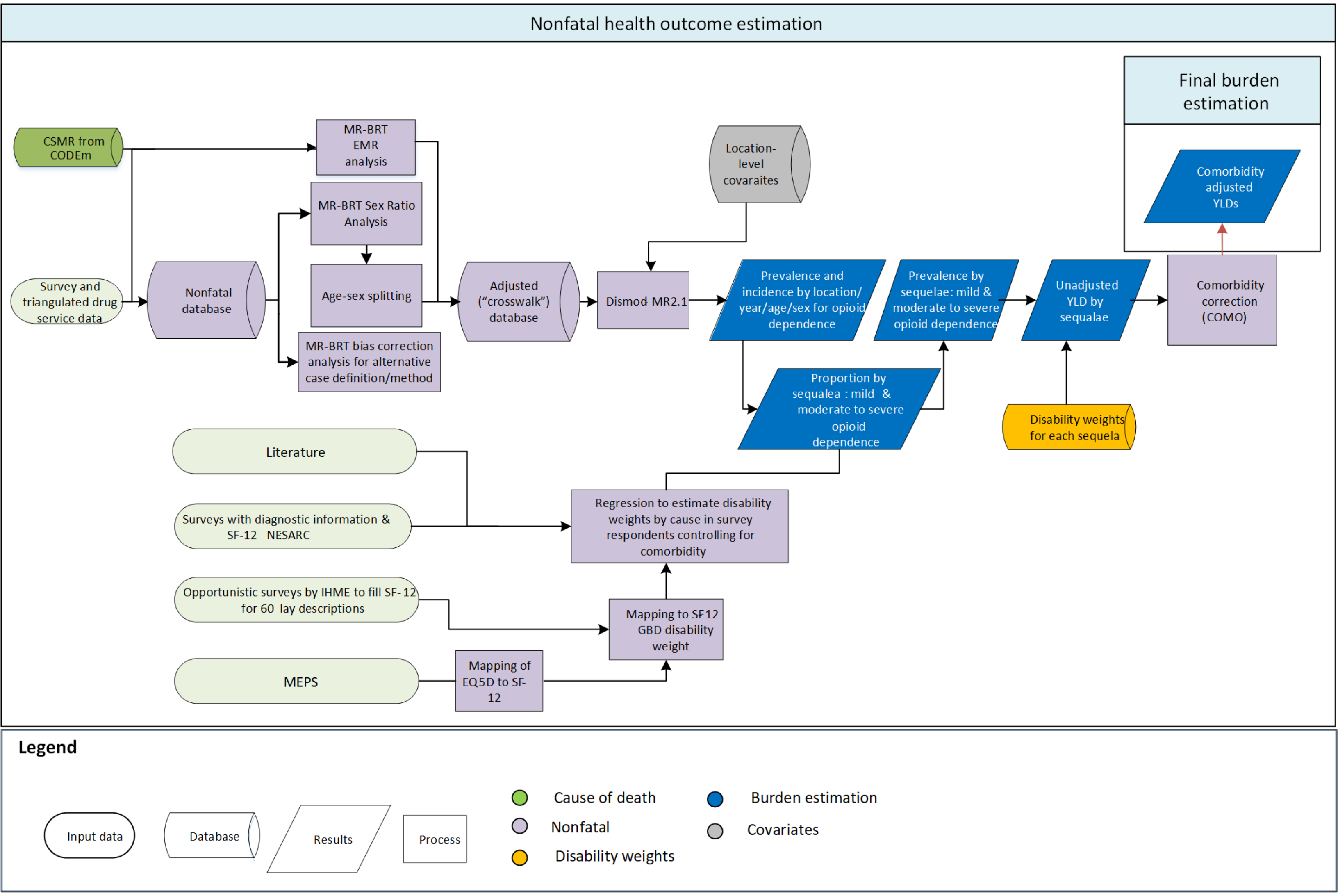

Figure S5 presents sex differences regarding DALYs by each SUD. Apart from Paraguay, all the countries had higher DALYs by SUDs (AmUD + CaUD + CoUD + OpUD) in males, compared to females

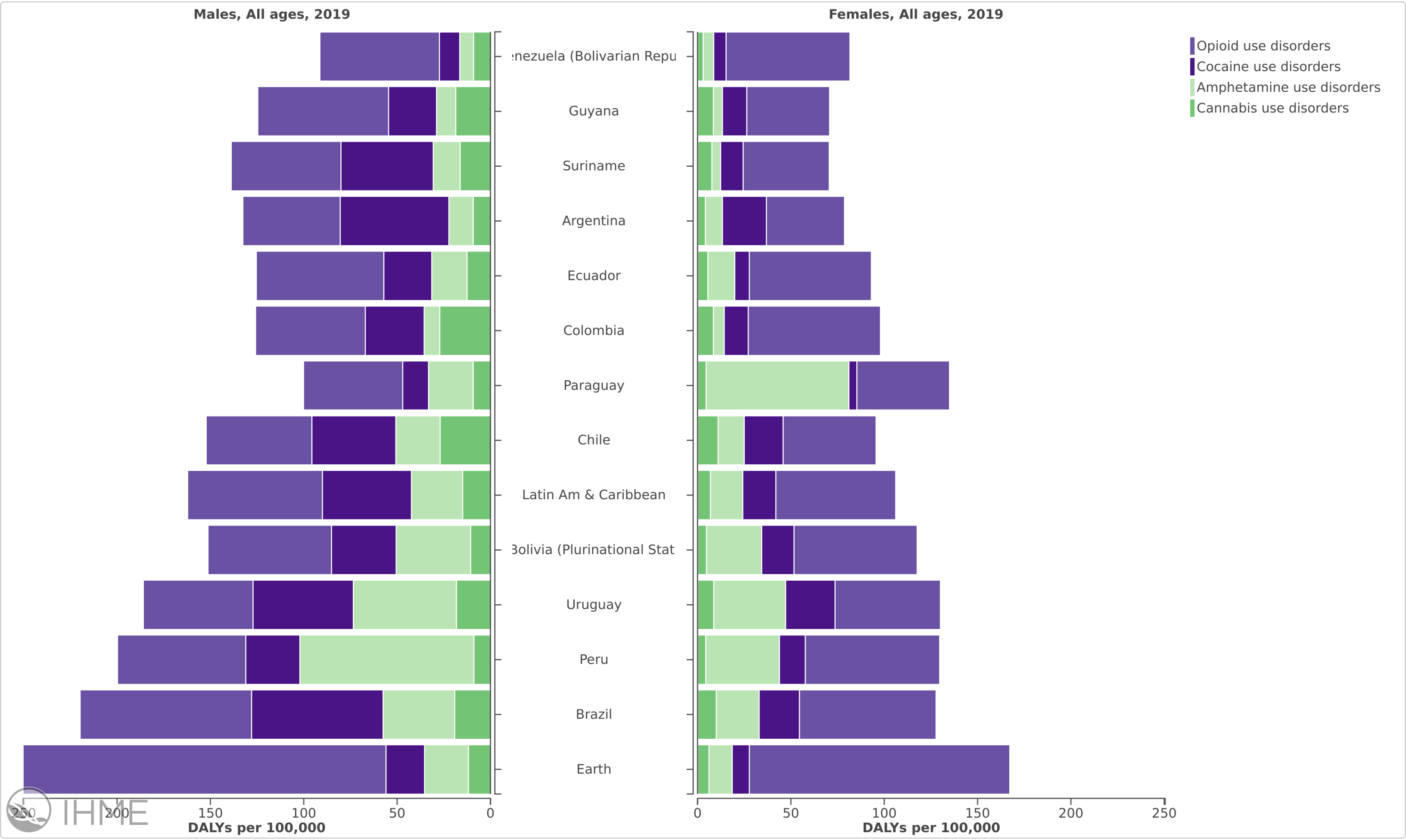

Table S1. Data quality rating from 0 to 5 stars, maximum percent well certified per 5-year interval and percent well certified across time series for South American countries, 1980-2019

Country	Data Quality Rating	1980-1984	1985-1989	1990-1994	1995-1999	2000-2004	2005-2009	2010-2019	1980-2019
Argentina	4	76.1	71.5	70.6	69.9	69.1	69.1	73.7	71.4
Bolivia	1	0.0	0.0	0.0	0.0	12.4	0.0	0.0	1.8
Brazil	4	51.3	55.5	62.0	66.2	70.9	77.8	82.3	66.6
Chile	4	75.3	75.9	82.0	85.1	91.2	91.5	90.4	84.5
Colombia	4	69.2	72.0	76.3	88.3	89.7	88.5	90.8	82.1
Ecuador	3	65.9	65.3	66.5	63.4	60.8	55.2	63.4	62.9
Guyana	4	49.2	65.9	61.4	68.1	73.5	75.1	74.2	66.8
Paraguay	3	51.7	54.5	61.5	62.3	63.1	67.2	82.2	63.2
Peru	3	51.6	34.4	34.5	45.8	55.4	56.2	52.1	47.1
Suriname	3	58.1	62.9	57.7	60.1	68.4	67.0	67.1	63.1
Uruguay	4	77.6	77.4	79.5	81.2	80.8	80.6	80.4	79.6
Venezuela	5	78.9	72.9	83.2	88.5	90.7	91.1	91.3	85.2

GBD data quality rating: the factors used in the computation of this rating are the percentage of fatalities allocated to specific GBD causes and the proportion of deaths not categorized to levels 1 or 2. Source: GBD 2019 Diseases and Injuries Collaborators. Global burden of 369 diseases and injuries in 204 countries and territories, 1990-2019: a systematic analysis for the Global Burden of Disease Study 2019. Lancet. 2020;396(10258):1204-1222.

Table S2. Underlying indicators for percent well-certified for data source with maximum percent well certified in each 5-year time interval for the South American countries, 1980-2019

Location	Time Window	Stars	Percent Well-Certified [PWC] (%)	Max PWC Data Year	Max PWC Data Source	Completeness (%)	Percent Major Garbage (%)
Argentina	1980-1984	4	76.1	1981	Vital Registration	100.0	23.9
Argentina	1985-1989	4	71.5	1988	Vital Registration	100.0	28.5
Argentina	1990-1994	4	70.6	1990	Vital Registration	100.0	29.4
Argentina	1995-1999	4	69.9	1996	Vital Registration	100.0	30.1
Argentina	2000-2004	4	69.1	2004	Vital Registration	100.0	30.9
Argentina	2005-2009	4	69.1	2005	Vital Registration	100.0	30.9
Argentina	2010-2019	4	73.7	2015	Vital Registration	100.0	26.3
Bolivia	1980-1984	0					
Bolivia	1985-1989	0					
Bolivia	1990-1994	0					
Bolivia	1995-1999	0					
Bolivia	2000-2004	2	12.4	2003	Vital Registration	38.1	67.3
Bolivia	2005-2009	0					
Bolivia	2010-2019	0					
Brazil	1980-1984	3	51.3	1984	Vital Registration	83.6	38.6
Brazil	1985-1989	3	55.5	1989	Vital Registration	85.2	34.8
Brazil	1990-1994	3	62.0	1994	Vital Registration	92.1	32.7
Brazil	1995-1999	4	66.2	1999	Vital Registration	93.6	29.3
Brazil	2000-2004	4	70.9	2004	Vital Registration	94.5	25.0
Brazil	2005-2009	4	77.8	2009	Vital Registration	96.1	19.0
Brazil	2010-2019	4	82.3	2014	Vital Registration	99.3	17.2
Chile	1980-1984	4	75.3	1983	Vital Registration	100.0	24.7
Chile	1985-1989	4	75.9	1988	Vital Registration	100.0	24.1
Chile	1990-1994	4	82.0	1994	Vital Registration	99.1	17.3
Chile	1995-1999	5	85.1	1999	Vital Registration	100.0	14.9

Chile	2000-2004	5	91.2	2003	Vital Registration	100.0	8.8
Chile	2005-2009	5	91.5	2006	Vital Registration	100.0	8.5
Chile	2010-2019	5	90.4	2012	Vital Registration	100.0	9.6
Colombia	1980-1984	4	69.2	1983	Vital Registration	92.4	25.0
Colombia	1985-1989	4	72.0	1987	Vital Registration	93.5	23.0
Colombia	1990-1994	4	76.3	1993	Vital Registration	93.5	18.4
Colombia	1995-1999	5	88.3	1999	Vital Registration	100.0	11.7
Colombia	2000-2004	5	89.7	2002	Vital Registration	100.0	10.3
Colombia	2005-2009	5	88.5	2008	Vital Registration	99.2	10.8
Colombia	2010-2019	5	90.8	2015	Vital Registration	100.0	9.2
Ecuador	1980-1984	4	65.9	1983	Vital Registration	99.9	34.0
Ecuador	1985-1989	4	65.3	1989	Vital Registration	95.0	31.3
Ecuador	1990-1994	4	66.5	1991	Vital Registration	94.4	29.5
Ecuador	1995-1999	3	63.4	1996	Vital Registration	90.4	29.9
Ecuador	2000-2004	3	60.8	2001	Vital Registration	91.0	33.2
Ecuador	2005-2009	3	55.2	2006	Vital Registration	75.8	27.2
Ecuador	2010-2019	3	63.4	2016	Vital Registration	74.2	14.6
Guyana	1980-1984	3	49.2	1984	Vital Registration	77.2	36.3
Guyana	1985-1989	4	65.9	1989	Vital Registration	88.0	25.0
Guyana	1990-1994	3	61.4	1990	Vital Registration	81.2	24.4
Guyana	1995-1999	4	68.1	1995	Vital Registration	87.7	22.4
Guyana	2000-2004	4	73.5	2004	Vital Registration	88.6	17.0
Guyana	2005-2009	4	75.1	2005	Vital Registration	88.8	15.5
Guyana	2010-2019	4	74.2	2010	Vital Registration	91.2	18.6
Paraguay	1980-1984	3	51.7	1984	Vital Registration	86.7	40.4
Paraguay	1985-1989	3	54.5	1989	Vital Registration	81.8	33.3
Paraguay	1990-1994	3	61.5	1994	Vital Registration	82.8	25.8
Paraguay	1995-1999	3	62.3	1996	Vital Registration	85.3	27.0

Paraguay	2000-2004	3	63.1	2004	Vital Registration	88.5	28.7
Paraguay	2005-2009	4	67.2	2009	Vital Registration	89.4	24.8
Paraguay	2010-2019	4	82.2	2016	Vital Registration	100.0	17.8
Peru	1980-1984	3	51.6	1980	Vital Registration	70.7	27.0
Peru	1985-1989	2	34.4	1989	Vital Registration	64.4	46.5
Peru	1990-1994	2	34.5	1992	Vital Registration	62.3	44.7
Peru	1995-1999	3	45.8	1999	Vital Registration	70.8	35.3
Peru	2000-2004	3	55.4	2004	Vital Registration	78.4	29.4
Peru	2005-2009	3	56.2	2007	Vital Registration	73.3	23.3
Peru	2010-2019	3	52.1	2015	Vital Registration	64.4	19.1
Suriname	1980-1984	3	58.1	1984	Vital Registration	90.0	35.4
Suriname	1985-1989	3	62.9	1989	Vital Registration	94.1	33.1
Suriname	1990-1994	3	57.7	1990	Vital Registration	82.1	29.8
Suriname	1995-1999	3	60.1	1999	Vital Registration	86.9	30.8
Suriname	2000-2004	4	68.4	2002	Vital Registration	89.4	23.4
Suriname	2005-2009	4	67.0	2008	Vital Registration	87.2	23.1
Suriname	2010-2019	4	67.1	2010	Vital Registration	85.0	21.0
Uruguay	1980-1984	4	77.6	1982	Vital Registration	100.0	22.4
Uruguay	1985-1989	4	77.4	1989	Vital Registration	99.4	22.1
Uruguay	1990-1994	4	79.5	1991	Vital Registration	100.0	20.5
Uruguay	1995-1999	4	81.2	1997	Vital Registration	99.4	18.4
Uruguay	2000-2004	4	80.8	2001	Vital Registration	100.0	19.2
Uruguay	2005-2009	4	80.6	2005	Vital Registration	100.0	19.4
Uruguay	2010-2019	4	80.4	2015	Vital Registration	100.0	19.6
Venezuela	1980-1984	4	78.9	1983	Vital Registration	100.0	21.1
Venezuela	1985-1989	4	72.9	1988	Vital Registration	99.3	26.5
Venezuela	1990-1994	4	83.2	1994	Vital Registration	100.0	16.8
Venezuela	1995-1999	5	88.5	1999	Vital Registration	100.0	11.5

Venezuela	2000-2004	5	90.7	2001	Vital Registration	100.0	9.3
Venezuela	2005-2009	5	91.1	2007	Vital Registration	100.0	8.9
Venezuela	2010-2019	5	91.3	2013	Vital Registration	99.8	8.5

Table S3. Drug dependence sequelae, health states, health state lay descriptions, and disability weights

Sequela	Health state name	Health state lay description	Disability Weight
Severe opioid dependence	Heroin and other opioid dependence	uses heroin daily and has difficulty controlling the habit. When the effects wear off, the person feels severe nausea, agitation, vomiting and fever. The person has a lot of difficulty in daily activities.	0.697 (0.51-0.843)
Mild opioid dependence	Heroin and other opioid dependence, mild	uses heroin (or methadone)Ã‚Â daily and has difficulty controlling the habit. When not using, the person functions normally.	0.335 (0.221-0.473)
Asymptomatic opioid dependence	Asymptomatic		0 (0-0)
Severe cocaine dependence	Cocaine dependence	uses cocaine and has difficulty controlling the habit. The person sometimes has mood swings, anxiety, paranoia, hallucinations and sleep problems, and has some difficulty in daily activities.	0.479 (0.324-0.634)
Mild cocaine dependence	Cocaine dependence, mild	uses cocaine at least once a week and has some difficulty controlling the habit. When not using, the person functions normally.	0.116 (0.074-0.165)
Asymptomatic cocaine dependence	Asymptomatic		0 (0-0)
Severe amphetamine dependence	Amphetamine dependence	uses stimulants (drugs) and has difficulty controlling the habit. The person sometimes has depression, hallucinations and mood swings, and has difficulty in daily activities.	0.486 (0.329-0.637)
Mild amphetamine dependence	Amphetamine dependence, mild	uses stimulants (drugs) at least once a week and has some difficulty controlling the habit. When not using, the person functions normally.	0.079 (0.051-0.114)
Asymptomatic amphetamine dependence	Asymptomatic		0 (0-0)
Severe cannabis dependence	Cannabis dependence	uses marijuana daily and has difficulty controlling the habit. The person sometimes has mood swings, anxiety and hallucinations, and has some difficulty in daily activities.	0.266 (0.178-0.364)
Mild cannabis dependence	Cannabis dependence, mild	uses marijuana at least once a week and has some difficulty controlling the habit. When not using, the person functions normally.	0.039 (0.024-0.06)
Asymptomatic cannabis dependence	Asymptomatic		0 (0-0)

Table S4. CODEm covariates used, level of covariate, and expected direction of covariate by cause, sex, and age

Covariates that CODEm did not select during the covariate selection process have no draw counts listed.

Cause	Sex	Age Start	Age End	Model Type	Direction	Level	Covariate Name	Number of Draws
Opioid use disorders	Female	15-19 years	95+ years	Data Rich	1	1	Opioids per million population per day (10 year lag)	190
Opioid use disorders	Female	15-19 years	95+ years	Data Rich	1	1	Opioids per million population per day	281
Opioid use disorders	Female	15-19 years	95+ years	Data Rich	1	1	Opioids per million population per day (5 year lag)	529
Opioid use disorders	Female	15-19 years	95+ years	Data Rich	0	2	Healthcare access and quality index	32
Opioid use disorders	Female	15-19 years	95+ years	Data Rich	1	2	Opium Cultivation (binary)	38
Opioid use disorders	Female	15-19 years	95+ years	Data Rich	1	2	Cumulative Cigarettes (10 Years)	--
Opioid use disorders	Female	15-19 years	95+ years	Data Rich	1	2	Cumulative Cigarettes (5 Years)	--
Opioid use disorders	Female	15-19 years	95+ years	Data Rich	1	2	Smoking Prevalence	--
Opioid use disorders	Female	15-19 years	95+ years	Data Rich	0	3	Socio-demographic Index	875
Opioid use disorders	Female	15-19 years	95+ years	Data Rich	0	3	Education (years per capita)	999
Opioid use disorders	Female	15-19 years	95+ years	Data Rich	0	3	LDI (I$ per capita)	--
Opioid use disorders	Female	15-19 years	95+ years	Global	1	1	Opioids per million population per day (10 year lag)	153

Opioid use disorders	Female	15-19 years	95+ years	Global	1	1	Opioids per million population per day (5 year lag)	332
Opioid use disorders	Female	15-19 years	95+ years	Global	1	1	Opioids per million population per day	515
Opioid use disorders	Female	15-19 years	95+ years	Global	0	2	Healthcare access and quality index	471
Opioid use disorders	Female	15-19 years	95+ years	Global	1	2	Opium Cultivation (binary)	25
Opioid use disorders	Female	15-19 years	95+ years	Global	1	2	Cumulative Cigarettes (10 Years)	--
Opioid use disorders	Female	15-19 years	95+ years	Global	1	2	Cumulative Cigarettes (5 Years)	--
Opioid use disorders	Female	15-19 years	95+ years	Global	1	2	Smoking Prevalence	--
Opioid use disorders	Female	15-19 years	95+ years	Global	0	3	Socio-demographic Index	700
Opioid use disorders	Female	15-19 years	95+ years	Global	0	3	Education (years per capita)	857
Opioid use disorders	Female	15-19 years	95+ years	Global	0	3	LDI (I$ per capita)	--
Opioid use disorders	Male	15-19 years	95+ years	Data Rich	1	1	Intravenous drug use (age-standardized proportion)	7
Opioid use disorders	Male	15-19 years	95+ years	Data Rich	1	1	Intravenous drug use (proportion by age)	833
Opioid use disorders	Male	15-19 years	95+ years	Data Rich	1	1	Opioids per million population per day (10 year lag)	1000
Opioid use disorders	Male	15-19 years	95+ years	Data Rich	0	2	Healthcare access and quality index	802

Opioid use disorders	Male	15-19 years	95+ years	Data Rich	1	2	Cumulative Cigarettes (10 Years)	--
Opioid use disorders	Male	15-19 years	95+ years	Data Rich	1	2	Cumulative Cigarettes (5 Years)	--
Opioid use disorders	Male	15-19 years	95+ years	Data Rich	1	2	Opium Cultivation (binary)	--
Opioid use disorders	Male	15-19 years	95+ years	Data Rich	1	2	Smoking Prevalence	--
Opioid use disorders	Male	15-19 years	95+ years	Data Rich	0	3	Socio-demographic Index	967
Opioid use disorders	Male	15-19 years	95+ years	Data Rich	0	3	Education (years per capita)	994
Opioid use disorders	Male	15-19 years	95+ years	Data Rich	0	3	LDI (I$ per capita)	--
Opioid use disorders	Male	15-19 years	95+ years	Global	1	1	Intravenous drug use (age-standardized proportion)	466
Opioid use disorders	Male	15-19 years	95+ years	Global	1	1	Intravenous drug use (proportion by age)	543
Opioid use disorders	Male	15-19 years	95+ years	Global	1	1	Opioids per million population per day (10 year lag)	997
Opioid use disorders	Male	15-19 years	95+ years	Global	0	2	Healthcare access and quality index	88
Opioid use disorders	Male	15-19 years	95+ years	Global	1	2	Opium Cultivation (binary)	0
Opioid use disorders	Male	15-19 years	95+ years	Global	1	2	Cumulative Cigarettes (10 Years)	--
Opioid use disorders	Male	15-19 years	95+ years	Global	1	2	Cumulative Cigarettes (5 Years)	--
Opioid use disorders	Male	15-19 years	95+ years	Global	1	2	Smoking Prevalence	--

Opioid use disorders	Male	15-19 years	95+ years	Global	0	3	Socio-demographic Index	571
Opioid use disorders	Male	15-19 years	95+ years	Global	0	3	Education (years per capita)	839
Opioid use disorders	Male	15-19 years	95+ years	Global	0	3	LDI (I$ per capita)	--
Cocaine use disorders	Female	15-19 years	95+ years	Data Rich	1	1	Alcohol (liters per capita)	1000
Cocaine use disorders	Female	15-19 years	95+ years	Data Rich	1	1	Cumulative Cigarettes (10 Years)	--
Cocaine use disorders	Female	15-19 years	95+ years	Data Rich	1	1	Cumulative Cigarettes (5 Years)	--
Cocaine use disorders	Female	15-19 years	95+ years	Data Rich	1	1	Smoking Prevalence	--
Cocaine use disorders	Female	15-19 years	95+ years	Data Rich	-1	2	Healthcare access and quality index	--
Cocaine use disorders	Female	15-19 years	95+ years	Data Rich	0	3	Education (years per capita)	926
Cocaine use disorders	Female	15-19 years	95+ years	Data Rich	0	3	LDI (I$ per capita)	--
Cocaine use disorders	Female	15-19 years	95+ years	Data Rich	1	3	Socio-demographic Index	--
Cocaine use disorders	Female	15-19 years	95+ years	Global	1	1	Alcohol (liters per capita)	1000
Cocaine use disorders	Female	15-19 years	95+ years	Global	1	1	Cumulative Cigarettes (10 Years)	--
Cocaine use disorders	Female	15-19 years	95+ years	Global	1	1	Cumulative Cigarettes (5 Years)	--
Cocaine use disorders	Female	15-19 years	95+ years	Global	1	1	Smoking Prevalence	--
Cocaine use disorders	Female	15-19 years	95+ years	Global	-1	2	Healthcare access and quality index	--
Cocaine use disorders	Female	15-19 years	95+ years	Global	0	3	Education (years per capita)	724

Cocaine use disorders	Female	15-19 years	95+ years	Global	0	3	LDI (I$ per capita)	--
Cocaine use disorders	Female	15-19 years	95+ years	Global	1	3	Socio-demographic Index	--
Cocaine use disorders	Male	15-19 years	95+ years	Data Rich	1	1	Alcohol (liters per capita)	193
Cocaine use disorders	Male	15-19 years	95+ years	Data Rich	1	1	Cumulative Cigarettes (10 Years)	--
Cocaine use disorders	Male	15-19 years	95+ years	Data Rich	1	1	Cumulative Cigarettes (5 Years)	--
Cocaine use disorders	Male	15-19 years	95+ years	Data Rich	1	1	Smoking Prevalence	--
Cocaine use disorders	Male	15-19 years	95+ years	Data Rich	-1	2	Healthcare access and quality index	--
Cocaine use disorders	Male	15-19 years	95+ years	Data Rich	0	3	Education (years per capita)	968
Cocaine use disorders	Male	15-19 years	95+ years	Data Rich	0	3	LDI (I$ per capita)	--
Cocaine use disorders	Male	15-19 years	95+ years	Data Rich	1	3	Socio-demographic Index	807
Cocaine use disorders	Male	15-19 years	95+ years	Global	1	1	Alcohol (liters per capita)	1000
Cocaine use disorders	Male	15-19 years	95+ years	Global	1	1	Cumulative Cigarettes (10 Years)	--
Cocaine use disorders	Male	15-19 years	95+ years	Global	1	1	Cumulative Cigarettes (5 Years)	--
Cocaine use disorders	Male	15-19 years	95+ years	Global	1	1	Smoking Prevalence	--
Cocaine use disorders	Male	15-19 years	95+ years	Global	-1	2	Healthcare access and quality index	--
Cocaine use disorders	Male	15-19 years	95+ years	Global	0	3	Education (years per capita)	724
Cocaine use disorders	Male	15-19 years	95+ years	Global	0	3	LDI (I$ per capita)	--

Cocaine use disorders	Male	15-19 years	95+ years	Global	1	3	Socio-demographic Index	449
Amphetamine use disorders	Female	15-19 years	95+ years	Data Rich	1	1	Alcohol (liters per capita)	166
Amphetamine use disorders	Female	15-19 years	95+ years	Data Rich	1	1	Cumulative Cigarettes (10 Years)	--
Amphetamine use disorders	Female	15-19 years	95+ years	Data Rich	1	1	Cumulative Cigarettes (5 Years)	--
Amphetamine use disorders	Female	15-19 years	95+ years	Data Rich	1	1	Smoking Prevalence	--
Amphetamine use disorders	Female	15-19 years	95+ years	Data Rich	-1	2	Healthcare access and quality index	--
Amphetamine use disorders	Female	15-19 years	95+ years	Data Rich	0	3	Education (years per capita)	834
Amphetamine use disorders	Female	15-19 years	95+ years	Data Rich	0	3	LDI (I$ per capita)	--
Amphetamine use disorders	Female	15-19 years	95+ years	Data Rich	1	3	Socio-demographic Index	834
Amphetamine use disorders	Female	15-19 years	95+ years	Global	1	1	Alcohol (liters per capita)	382
Amphetamine use disorders	Female	15-19 years	95+ years	Global	1	1	Cumulative Cigarettes (10 Years)	--
Amphetamine use disorders	Female	15-19 years	95+ years	Global	1	1	Cumulative Cigarettes (5 Years)	--
Amphetamine use disorders	Female	15-19 years	95+ years	Global	1	1	Smoking Prevalence	--
Amphetamine use disorders	Female	15-19 years	95+ years	Global	-1	2	Healthcare access and quality index	--
Amphetamine use disorders	Female	15-19 years	95+ years	Global	0	3	Education (years per capita)	618
Amphetamine use disorders	Female	15-19 years	95+ years	Global	0	3	LDI (I$ per capita)	--
Amphetamine use disorders	Female	15-19 years	95+ years	Global	1	3	Socio-demographic Index	618

Amphetamine use disorders	Male	15-19 years	95+ years	Data Rich	1	1	Alcohol (liters per capita)	1000
Amphetamine use disorders	Male	15-19 years	95+ years	Data Rich	1	1	Cumulative Cigarettes (10 Years)	--
Amphetamine use disorders	Male	15-19 years	95+ years	Data Rich	1	1	Cumulative Cigarettes (5 Years)	--
Amphetamine use disorders	Male	15-19 years	95+ years	Data Rich	1	1	Smoking Prevalence	--
Amphetamine use disorders	Male	15-19 years	95+ years	Data Rich	-1	2	Healthcare access and quality index	--
Amphetamine use disorders	Male	15-19 years	95+ years	Data Rich	0	3	Education (years per capita)	--
Amphetamine use disorders	Male	15-19 years	95+ years	Data Rich	0	3	LDI (I$ per capita)	--
Amphetamine use disorders	Male	15-19 years	95+ years	Data Rich	1	3	Socio-demographic Index	--
Amphetamine use disorders	Male	15-19 years	95+ years	Global	1	1	Alcohol (liters per capita)	1000
Amphetamine use disorders	Male	15-19 years	95+ years	Global	1	1	Cumulative Cigarettes (10 Years)	--
Amphetamine use disorders	Male	15-19 years	95+ years	Global	1	1	Cumulative Cigarettes (5 Years)	--
Amphetamine use disorders	Male	15-19 years	95+ years	Global	1	1	Smoking Prevalence	--
Amphetamine use disorders	Male	15-19 years	95+ years	Global	-1	2	Healthcare access and quality index	--
Amphetamine use disorders	Male	15-19 years	95+ years	Global	0	3	Education (years per capita)	500
Amphetamine use disorders	Male	15-19 years	95+ years	Global	0	3	LDI (I$ per capita)	--
Amphetamine use disorders	Male	15-19 years	95+ years	Global	1	3	Socio-demographic Index	--

Table S5. CODEm predictive validity results by cause, model type, sex, and age									
**Cause**	**Sex**	**Age Start**	**Age End**	**RMSE In-Sample**	**RMSE Out-of-Sample**	**Trend In-Sample**	**Trend Out-of-Sample**	**Coverage In-Sample**	**Coverage Out-of-Sample**
Opioid use disorders [Data Rich]	Female	15-19 years	95+ years	0.186057	0.456549	0.132543	0.204713	0.999962	0.999626
Opioid use disorders [Global]	Female	15-19 years	95+ years	0.260574	0.596691	0.181748	0.196987	0.999352	0.991017
Opioid use disorders [Data Rich]	Male	15-19 years	95+ years	0.242361	0.478018	0.152908	0.183021	0.999922	0.999829
Opioid use disorders [Global]	Male	15-19 years	95+ years	0.259563	0.572114	0.16524	0.18041	0.999753	0.993191
Cocaine use disorders [Data Rich]	Female	15-19 years	95+ years	0.299559	0.63854	0.21168	0.248099	0.997853	0.996335
Cocaine use disorders [Global]	Female	15-19 years	95+ years	0.391245	0.821799	0.267302	0.280228	0.995261	0.978889
Cocaine use disorders [Data Rich]	Male	15-19 years	95+ years	0.286565	0.585871	0.175354	0.208299	0.999229	0.998421
Cocaine use disorders [Global]	Male	15-19 years	95+ years	0.356246	0.770239	0.219514	0.235481	0.997929	0.9856
Amphetamine use disorders [Global]	Male	15-19 years	95+ years	0.35858	0.739732	0.215124	0.225448	0.998164	0.98664
Amphetamine use disorders [Data Rich]	Male	15-19 years	95+ years	0.263658	0.746558	0.164977	0.210133	0.999465	0.996506
Amphetamine use disorders [Data Rich]	Female	15-19 years	95+ years	0.26707	0.88871	0.182591	0.2053	0.998767	0.996239
Amphetamine use disorders [Global]	Female	15-19 years	95+ years	0.363582	0.806592	0.237206	0.247872	0.997427	0.980178

Table S6. All-age and age standardized DALYs rates per 100,000 individuals by location for 1990, 2010, and 2019, both sexes combined

	All-age DALYs per 100,000												Age-standardized DALYs per 100,000											
	1990			2000			2010			2019			1990			2000			2010			2019		
	Prev	95%UI		Prev	95%UI		Prev	95%UI		Prev	95%UI		Prev	95%UI		Prev	95%UI		Prev	95%UI		Prev	95%UI	
**Amphetamine Use Disorder**																								
Global	24.51	36.36	16.29	24.02	34.06	17.07	18.04	26.96	11.74	18.08	26.36	12.34	23.26	34.33	15.58	22.99	32.50	16.40	17.08	25.58	11.14	17.54	25.58	11.93
Latin America and Caribbean	21.26	35.12	11.65	21.56	35.02	11.98	22.10	35.90	12.43	22.27	36.63	12.57	19.95	33.07	11.03	20.00	32.35	11.18	20.31	33.06	11.50	20.96	34.46	11.81
Argentina	10.40	17.31	5.49	10.91	18.99	6.04	9.66	15.48	5.51	11.08	18.57	5.93	10.80	17.95	5.69	10.84	18.65	5.99	9.31	14.90	5.31	10.73	18.07	5.75
Bolivia	30.21	49.41	16.40	31.60	52.69	16.90	35.51	58.48	19.74	34.75	57.67	19.51	31.12	50.93	16.95	31.41	51.69	17.17	33.08	54.24	18.56	32.82	53.97	18.75
Brazil	32.18	53.73	17.06	32.31	53.92	17.39	32.99	54.55	17.95	30.56	52.00	16.77	29.41	49.43	15.73	29.05	48.40	15.68	29.39	48.59	16.04	28.91	49.35	15.80
Chile	21.63	37.02	11.20	16.26	26.57	9.05	20.26	33.14	11.66	18.80	30.87	10.73	19.11	32.38	10.02	15.45	25.36	8.54	19.18	31.33	10.98	18.16	29.96	10.30
Colombia	8.35	13.26	5.00	6.72	11.22	3.60	6.68	10.91	3.70	7.11	11.64	4.09	7.70	11.87	4.83	6.41	10.64	3.50	6.41	10.42	3.59	6.61	10.84	3.82
Ecuador	15.51	26.74	8.04	15.62	26.38	8.24	18.51	30.57	9.69	16.60	27.74	9.00	14.74	25.08	7.87	14.68	24.61	7.84	17.38	28.49	9.24	15.41	25.53	8.41
Guyana	6.83	12.21	3.29	7.63	13.19	4.09	6.42	10.81	3.56	7.62	12.48	4.19	6.02	10.64	3.04	7.06	12.02	3.83	6.39	10.77	3.55	6.71	10.89	3.78
Paraguay	42.72	71.10	22.11	42.68	71.66	22.76	50.66	83.84	26.57	49.93	83.28	26.76	43.05	72.00	22.86	42.81	71.56	22.89	45.65	74.54	24.27	44.35	73.74	23.88
Peru	61.67	102.36	32.42	64.07	105.78	35.04	68.20	111.07	37.09	66.47	109.72	36.56	59.55	99.16	31.84	59.89	98.69	32.86	62.98	102.09	34.67	62.55	103.04	34.56
Suriname	7.21	11.86	3.73	10.79	15.80	7.24	9.44	14.05	5.98	9.41	13.83	6.33	6.53	10.57	3.50	10.12	14.60	6.86	8.89	13.19	5.63	9.30	13.74	6.20
Uruguay	45.74	76.19	24.50	48.84	80.87	27.34	52.25	86.70	28.54	46.56	77.99	26.28	47.62	79.18	25.44	50.28	83.82	28.17	54.22	90.17	29.46	48.44	81.67	26.97
Venezuela	6.63	11.50	3.44	6.77	11.46	3.66	7.17	11.69	3.93	6.62	10.56	3.76	6.15	10.48	3.27	6.29	10.47	3.47	6.50	10.52	3.66	6.64	10.64	3.69
**Cannabis Use Disorder**																								
Global	9.31	14.59	5.56	8.95	13.97	5.41	8.91	13.85	5.39	8.92	13.92	5.44	8.78	13.67	5.29	8.50	13.26	5.17	8.42	13.12	5.09	8.79	13.68	5.32
Latin America and Caribbean	11.03	17.94	6.35	11.34	17.66	6.71	11.17	17.42	6.66	10.85	16.66	6.49	10.26	16.47	6.04	10.39	15.97	6.20	10.29	15.96	6.18	10.34	15.91	6.19
Argentina	6.45	10.11	3.71	6.72	10.09	4.16	6.76	9.99	4.23	6.59	9.75	4.13	6.52	10.15	3.73	6.49	9.71	4.00	6.53	9.60	4.08	6.58	9.74	4.14
Bolivia	6.85	10.63	3.89	7.57	11.70	4.52	7.97	12.46	4.55	7.66	11.92	4.55	6.97	10.83	4.02	7.31	11.19	4.35	7.35	11.33	4.26	7.38	11.49	4.43
Brazil	15.74	25.86	8.96	15.64	24.69	9.11	15.31	24.14	9.01	14.44	22.39	8.55	14.46	23.43	8.39	13.96	21.78	8.20	13.94	21.89	8.24	13.88	21.69	8.18
Chile	12.85	19.27	7.92	16.32	23.87	10.51	17.18	25.08	11.10	18.84	27.54	12.21	11.39	16.98	7.07	15.63	22.90	10.05	16.35	23.91	10.57	19.68	28.75	12.75
Colombia	9.73	15.18	5.73	16.17	24.13	9.93	16.11	24.19	10.03	17.58	26.39	11.12	8.81	13.52	5.21	14.91	22.28	9.22	15.05	22.64	9.37	16.76	25.11	10.57
Ecuador	9.05	14.54	4.96	9.00	14.05	5.21	8.99	14.22	5.08	9.09	14.54	5.17	8.54	13.59	4.78	8.42	13.05	4.89	8.46	13.29	4.89	8.48	13.55	4.88
Guyana	13.08	21.19	7.08	12.27	20.02	6.93	12.43	19.91	7.11	13.47	23.57	6.95	11.39	18.18	6.30	11.44	18.58	6.47	11.45	18.40	6.53	11.88	20.61	6.26
Paraguay	5.86	9.21	3.32	6.13	9.47	3.42	6.55	10.29	3.73	6.94	11.23	3.93	5.96	9.10	3.42	5.96	9.18	3.34	5.96	9.29	3.42	6.33	10.10	3.65
Peru	6.88	11.30	3.73	6.75	10.69	3.79	6.79	10.67	3.84	6.62	10.45	3.69	6.54	10.66	3.64	6.29	9.85	3.57	6.29	9.86	3.58	6.31	9.97	3.53
Suriname	14.06	24.92	7.23	13.22	21.98	7.44	12.63	21.11	7.07	11.96	19.80	6.74	12.54	21.76	6.62	11.98	19.78	6.82	11.94	19.89	6.66	11.89	19.90	6.69
Uruguay	13.81	21.77	7.88	14.13	21.00	8.56	13.98	20.84	8.63	13.24	20.03	8.00	14.27	22.50	8.15	14.55	21.60	8.84	14.57	21.71	9.01	14.25	21.66	8.57
Venezuela	6.64	11.01	3.69	6.18	10.07	3.46	6.42	10.15	3.60	5.91	9.26	3.43	6.32	10.28	3.59	5.83	9.34	3.32	5.87	9.30	3.30	5.80	9.17	3.33
**Cocaine Use Disorder**																								
Global	10.06	13.95	6.96	11.72	15.38	8.68	12.63	16.35	9.58	14.90	18.50	12.06	10.04	13.79	6.93	11.51	15.04	8.53	12.10	15.66	9.18	14.29	17.77	11.54
Latin America and Caribbean	22.51	31.39	15.47	28.03	38.13	19.90	31.91	43.73	22.76	32.44	42.69	24.42	22.30	30.71	15.70	26.93	36.37	19.30	29.86	40.63	21.41	30.75	40.48	23.13
Argentina	34.36	52.68	19.79	34.18	50.40	20.77	44.82	63.69	29.52	40.48	60.85	24.17	35.19	54.00	20.23	33.74	49.66	20.55	43.31	61.48	28.54	39.13	59.16	23.34
Bolivia	20.95	30.31	13.96	26.02	37.35	18.00	28.91	41.51	19.46	26.01	37.09	17.23	22.25	31.46	15.35	26.34	37.06	18.73	27.63	39.61	18.95	26.00	36.47	17.51
Brazil	22.31	32.69	14.44	29.14	42.70	18.68	43.73	60.92	30.34	45.43	59.25	34.37	21.93	31.68	14.57	27.01	38.84	17.47	40.00	55.65	27.77	42.83	56.00	32.37
Chile	27.82	42.79	15.92	35.63	51.90	23.13	37.93	55.22	23.90	32.63	49.03	19.31	25.86	39.44	15.07	34.00	49.44	22.06	35.37	51.69	22.24	31.65	48.65	18.49
Colombia	32.34	43.98	23.34	23.59	35.16	14.43	27.93	40.20	18.52	21.97	31.74	14.09	30.68	40.87	22.56	22.44	33.13	14.04	26.39	37.80	17.52	20.69	30.06	13.24
Ecuador	12.73	18.04	8.77	13.61	19.52	9.34	15.97	21.72	11.50	16.65	22.52	12.09	13.00	17.85	9.38	13.36	18.78	9.45	15.57	20.77	11.37	15.96	21.52	11.69
Guyana	13.58	21.88	7.45	17.58	26.26	11.38	16.41	24.68	10.30	19.20	27.89	12.39	12.42	19.41	7.07	16.99	25.00	11.13	15.73	23.62	10.10	17.52	25.05	11.37
Paraguay	4.83	7.55	2.82	5.52	8.49	3.35	7.09	10.43	4.67	8.97	12.15	6.17	5.06	7.71	3.08	5.52	8.39	3.40	6.64	9.50	4.47	8.35	11.24	5.82
Peru	17.39	23.41	12.55	21.94	29.04	16.74	26.56	34.36	20.67	21.41	28.56	15.69	17.19	22.78	12.69	20.98	27.45	16.14	25.00	32.14	19.54	20.56	27.43	15.11
Suriname	18.18	27.31	11.23	37.73	49.32	28.61	30.44	41.02	22.41	30.58	40.55	22.58	17.16	25.19	10.85	35.65	46.02	27.29	28.77	38.81	21.18	30.00	40.21	22.08
Uruguay	34.24	53.82	19.70	36.05	54.10	21.33	42.75	62.79	26.46	39.50	59.48	23.74	34.92	55.12	20.08	36.43	54.93	21.46	43.19	63.33	26.60	40.07	60.90	23.86
Venezuela	6.10	8.46	4.30	6.96	9.32	5.08	8.59	11.25	6.59	8.66	11.41	6.41	6.15	8.25	4.51	6.75	8.80	5.06	8.07	10.43	6.28	8.46	11.21	6.26
**Opioid Use Disorder**																								
Global	116.59	152.33	88.66	141.80	179.41	110.54	145.40	185.18	111.42	167.07	209.63	129.26	118.23	151.44	90.76	139.66	175.40	109.33	139.59	177.15	107.10	160.37	201.67	123.96
Latin America and Caribbean	51.20	72.46	34.49	56.89	79.16	38.68	61.28	85.29	41.46	68.08	93.16	46.85	54.87	75.49	37.29	58.14	79.26	40.19	59.38	81.69	40.62	64.07	87.53	44.16
Argentina	31.20	43.50	21.19	37.87	52.11	25.83	41.86	57.63	28.69	46.79	63.84	32.22	32.43	45.07	22.08	38.15	52.55	26.03	40.48	55.78	27.76	43.88	60.02	30.09
Bolivia	67.98	97.87	44.75	71.88	102.65	47.84	62.73	89.34	41.53	66.00	92.61	44.10	81.65	114.69	55.40	83.03	116.09	56.64	67.33	93.09	45.93	68.87	95.35	46.64
Brazil	53.61	76.44	35.04	58.78	83.01	38.50	70.70	100.18	47.17	82.28	113.06	55.47	55.38	77.12	36.63	57.88	80.68	38.62	65.30	91.49	43.74	75.48	103.79	50.85
Chile	42.41	57.90	29.06	39.73	55.21	26.90	47.39	64.07	33.66	53.22	70.37	38.56	41.24	55.96	28.47	38.23	52.97	26.06	43.72	59.58	31.06	48.57	64.95	35.03
Colombia	58.86	84.41	38.83	55.33	79.27	36.21	58.42	83.69	38.20	64.96	91.98	42.82	60.75	85.17	41.02	56.50	80.08	37.09	57.63	82.34	37.63	60.33	85.72	39.75
Ecuador	42.74	61.84	27.78	50.27	71.43	33.55	61.20	86.29	40.88	66.85	93.16	44.83	49.08	68.40	32.67	54.38	75.11	37.24	63.62	88.52	43.24	66.44	91.42	45.19
Guyana	39.73	55.50	26.32	49.88	68.27	33.95	47.41	64.62	32.72	57.15	77.55	40.31	42.23	56.97	28.54	51.05	69.35	35.00	48.55	65.88	33.70	54.30	73.05	38.65
Paraguay	45.70	67.37	29.04	46.98	68.17	29.89	48.45	69.62	31.59	51.49	73.38	33.81	52.72	74.63	34.21	52.49	74.19	34.06	49.10	69.30	32.28	49.15	69.06	32.42
Peru	45.67	63.95	30.47	55.15	76.77	38.11	69.22	95.30	47.49	70.30	97.10	47.60	50.62	69.07	34.33	57.53	78.32	39.99	68.08	92.99	47.13	67.62	93.12	46.11
Suriname	33.87	48.92	21.73	46.41	62.91	32.94	48.32	66.39	32.39	52.32	71.42	35.42	35.10	49.25	23.38	45.41	60.78	32.57	46.19	63.06	31.21	50.78	69.49	34.74
Uruguay	45.99	66.27	29.80	54.10	75.06	37.32	54.14	72.50	37.77	57.55	76.79	41.29	46.00	66.75	29.66	53.19	74.86	36.25	52.00	70.36	35.92	54.27	73.60	38.53
Venezuela	67.37	100.58	41.80	70.61	101.64	45.32	69.35	98.28	45.73	65.31	90.67	44.40	71.80	104.72	46.01	71.73	101.99	46.20	66.48	93.68	44.07	63.19	87.96	43.11

Table S7. All-age and age standardized prevalence rates per 100,000 individuals by location for 1990, 2010, and 2019, both sexes combined

	All-age prevalence per 100,000												Age-standardized prevalence per 100,000											
	1990			2000			2010			2019			1990			2000			2010			2019		
	Prev	95%UI		Prev	95%UI		Prev	95%UI		Prev	95%UI		Prev	95%UI		Prev	95%UI		Prev	95%UI		Prev	95%UI	
**Amphetamine Use Disorder**																								
Global	135	89	189	119	163	81	106	146	71	95	131	63	127	176	85	113	154	77	100	137	67	93	128	62
Latin America and Caribbean	157	102	226	156	221	104	161	226	106	160	226	105	146	209	96	144	203	97	148	207	98	151	213	99
Argentina	78	50	108	81	114	52	71	91	53	81	113	52	81	112	52	81	112	52	68	88	51	79	110	50
Bolivia	225	145	319	233	323	152	261	370	169	253	358	165	231	323	153	230	317	153	242	342	159	238	335	157
Brazil	246	157	355	247	349	160	251	354	164	231	326	151	225	321	147	222	313	146	223	314	147	219	308	142
Chile	163	105	232	122	160	90	149	204	105	137	190	92	144	203	95	116	152	86	141	193	98	132	186	88
Colombia	50	31	72	47	67	30	46	65	29	47	68	30	44	62	29	44	62	28	44	62	28	44	63	28
Ecuador	113	67	166	114	169	68	133	195	84	117	171	71	106	153	65	106	156	65	124	180	80	108	157	66
Guyana	51	30	75	53	79	33	45	66	28	52	75	32	45	65	28	49	71	31	45	66	28	45	65	28
Paraguay	326	210	456	326	461	211	386	536	251	379	532	255	330	456	215	328	457	215	348	483	228	337	470	231
Peru	460	311	652	473	651	307	500	684	334	491	683	326	444	629	304	442	610	288	461	629	312	462	642	308
Suriname	51	31	74	53	77	34	51	72	33	47	67	30	46	65	29	49	69	31	48	68	32	47	68	30
Uruguay	348	237	481	369	511	250	394	547	273	350	485	243	362	502	245	379	528	256	409	564	282	364	509	250
Venezuela	48	29	68	48	69	30	49	70	30	43	62	28	44	62	28	44	62	28	44	63	28	44	63	28
**Cannabis Use Disorder**																								
Global	321	426	239	309	403	234	308	401	233	308	399	231	303	397	227	294	379	222	291	378	220	303	396	226
Latin America and Caribbean	381	530	268	391	515	290	385	502	288	375	486	281	355	482	258	359	466	270	355	460	267	357	463	268
Argentina	221	289	166	230	270	196	231	269	197	227	268	193	224	292	168	222	260	190	223	260	190	227	269	192
Bolivia	236	312	173	260	336	196	274	353	207	263	338	199	240	311	181	252	323	193	253	325	194	253	325	194
Brazil	546	773	379	543	727	390	530	703	385	501	654	368	502	696	357	485	641	353	483	638	352	481	635	351
Chile	442	530	368	562	619	509	592	650	533	648	717	582	392	468	328	539	592	488	563	619	506	676	750	606
Colombia	334	436	257	556	667	464	553	663	463	605	730	504	303	388	237	513	616	431	517	621	434	577	696	480
Ecuador	311	428	211	309	411	221	308	409	220	312	414	224	294	397	206	290	383	210	290	384	210	291	385	211
Guyana	452	631	311	424	587	295	429	590	304	465	704	298	395	541	281	396	543	282	396	542	282	411	611	268
Paraguay	202	271	145	211	283	151	226	304	162	239	331	168	205	271	151	205	271	151	206	272	151	218	297	155
Peru	235	317	164	232	311	166	233	312	169	228	303	166	224	297	162	216	287	158	216	288	158	217	289	159
Suriname	484	761	300	456	661	309	435	621	301	413	584	288	432	659	277	413	590	285	411	587	284	410	586	283
Uruguay	474	629	338	486	602	392	480	594	388	455	570	360	490	652	348	500	621	404	500	620	404	489	612	386
Venezuela	227	325	154	213	295	152	220	305	160	203	273	151	217	301	153	200	274	147	202	275	147	199	273	146
**Cocaine Use Disorder**																								
Global	58	76	44	57	73	45	58	73	47	53	67	42	57	74	44	56	71	45	56	70	45	52	65	41
Latin America and Caribbean	119	164	85	140	191	102	164	221	123	141	190	104	116	159	86	133	180	99	153	204	116	134	181	99
Argentina	250	329	185	247	311	196	324	366	286	292	377	222	256	338	190	244	308	194	313	354	276	282	366	214
Bolivia	106	147	72	128	180	89	146	202	102	120	164	85	109	148	77	126	173	90	135	184	96	117	158	84
Brazil	137	190	99	182	253	131	242	328	178	201	268	152	134	182	99	169	231	123	222	300	164	190	256	142
Chile	201	265	148	256	297	218	267	325	220	227	303	170	187	243	139	244	283	209	249	304	205	221	295	162
Colombia	144	200	99	146	200	103	175	215	141	121	162	87	134	182	95	139	187	99	165	203	134	114	153	82
Ecuador	57	81	39	64	88	44	68	94	47	64	88	45	55	76	39	61	82	44	65	89	46	61	82	43
Guyana	98	136	67	95	132	66	97	133	69	103	141	72	90	122	64	92	126	65	93	125	67	93	127	66
Paraguay	29	41	20	32	47	22	35	50	25	34	47	24	30	42	22	32	45	23	32	45	23	31	43	22
Peru	67	94	46	71	100	50	74	102	53	66	91	47	64	88	47	66	92	48	69	94	49	63	87	45
Suriname	112	156	78	135	184	96	127	169	91	117	160	86	104	142	75	125	169	90	121	161	87	116	159	85
Uruguay	248	339	183	256	347	192	299	380	231	270	359	200	252	346	186	258	353	192	301	386	232	273	369	200
Venezuela	25	36	17	26	39	18	29	41	20	25	35	18	24	33	17	25	35	17	26	37	19	25	35	18
**Opioid Use Disorder**																								
Global	201	263	159	229	292	186	248	315	200	276	348	226	203	261	163	226	285	184	239	301	194	266	334	217
Latin America and Caribbean	116	154	89	127	164	99	140	179	109	155	197	123	125	160	98	131	164	103	136	171	108	146	185	116
Argentina	71	91	56	84	107	66	92	117	72	103	131	83	74	95	58	85	108	67	88	112	70	97	124	77
Bolivia	156	211	119	164	219	125	141	184	111	149	190	117	188	244	147	190	245	149	152	192	121	156	197	125
Brazil	127	169	97	139	182	108	167	217	129	194	247	153	132	170	102	138	176	108	154	199	120	177	227	140
Chile	94	116	76	87	111	68	89	116	69	101	130	78	92	112	76	84	106	66	83	109	63	91	119	70
Colombia	130	174	98	126	168	95	134	175	102	149	194	115	135	173	104	130	170	100	132	172	101	138	181	106
Ecuador	95	128	70	114	149	88	140	181	110	152	196	120	109	141	83	123	157	97	146	185	116	151	192	121
Guyana	91	118	70	114	144	93	109	133	89	128	158	105	98	123	77	118	146	98	112	136	92	122	149	102
Paraguay	107	145	79	109	144	80	111	148	82	117	154	87	125	163	94	122	162	91	113	148	84	112	146	84
Peru	101	130	79	121	154	95	153	195	121	158	201	126	112	141	90	126	159	102	151	189	121	152	192	122
Suriname	78	103	59	95	121	76	106	135	85	116	145	93	81	104	62	94	117	76	102	129	81	112	142	90
Uruguay	105	138	79	114	149	86	109	141	84	114	146	89	105	139	78	112	149	83	104	138	79	107	141	81
Venezuela	158	214	116	165	217	124	158	203	123	150	189	119	169	222	128	168	216	129	152	193	119	145	184	114

Table S8. All-age and age standardized incidence rates per 100,000 individuals by location for 1990, 2010, and 2019, both sexes combined

	All-age incidence per 100,000												Age-standardized incidence per 100,000											
	1990			2000			2010			2019			1990			2000			2010			2019		
	Inc	95%UI		Inc	95%UI		Inc	95%UI		Inc	95%UI		Inc	95%UI		Inc	95%UI		Inc	95%UI		Inc	95%UI	
**Amphetamine Use Disorder**																								
Global	21.74	29.42	15.01	18.69	25.04	13.21	17.18	23.11	12.22	14.58	19.54	10.35	19.69	26.47	13.77	17.58	23.48	12.55	16.05	21.49	11.49	14.49	19.53	10.24
Latin America and Caribbean	25.11	34.76	16.21	25.24	35.19	16.61	25.20	34.17	16.91	23.84	32.37	15.80	21.98	29.97	14.67	22.20	30.48	14.84	22.71	30.86	15.47	22.73	30.90	15.04
Argentina	16.02	22.18	10.93	16.90	23.49	11.26	14.96	19.10	11.08	16.38	22.39	11.06	16.37	22.53	11.18	16.41	22.65	11.06	14.38	18.32	10.64	16.10	22.20	10.76
Bolivia	37.51	52.15	24.00	39.31	55.56	24.98	43.15	59.90	28.20	39.01	54.15	24.96	35.29	48.09	23.26	35.30	49.26	23.00	37.13	51.02	24.85	36.20	49.86	23.43
Brazil	38.07	52.74	24.59	39.45	54.59	26.06	37.85	51.67	25.76	32.90	44.47	22.32	33.36	45.47	22.05	33.82	46.34	22.64	34.14	46.48	23.33	32.36	43.91	21.56
Chile	30.67	42.30	20.58	23.25	30.42	17.08	28.45	38.11	20.48	24.41	32.77	17.23	26.73	36.66	18.28	22.39	29.34	16.45	26.89	36.22	19.26	24.85	33.80	17.36
Colombia	10.30	14.62	6.65	9.66	13.68	6.31	9.48	13.43	6.14	9.45	13.14	6.24	8.95	12.50	5.96	8.92	12.58	5.92	8.89	12.51	5.81	8.87	12.33	5.85
Ecuador	21.68	30.72	14.01	21.61	30.82	13.43	24.60	34.40	15.98	21.48	30.66	13.95	19.27	26.92	12.59	19.29	27.30	12.37	22.38	30.95	14.80	19.49	27.49	12.75
Guyana	9.82	13.77	6.20	9.76	13.70	6.26	8.80	12.44	5.79	9.79	13.57	6.40	8.29	11.31	5.48	8.93	12.41	5.82	8.30	11.62	5.58	8.38	11.57	5.56
Paraguay	48.97	67.99	30.73	50.76	71.07	32.04	58.38	81.53	37.94	52.91	74.05	34.31	45.66	62.95	29.30	45.40	63.29	29.54	48.48	67.03	31.89	46.58	64.81	30.39
Peru	65.44	92.14	42.47	65.36	92.78	42.69	65.81	89.30	44.47	60.53	83.27	40.04	55.94	76.94	37.22	55.99	79.08	37.11	58.30	78.55	39.87	57.51	79.04	38.07
Suriname	9.74	13.39	6.32	9.77	13.54	6.63	9.10	12.42	6.29	8.63	11.79	5.98	8.41	11.31	5.64	8.81	12.10	6.08	8.62	11.72	5.96	8.65	11.82	5.95
Uruguay	50.23	68.74	34.05	53.74	72.54	36.24	57.79	78.09	39.86	49.16	67.44	33.87	52.04	71.23	35.16	54.98	74.31	36.99	60.53	82.00	41.40	52.38	72.01	35.69
Venezuela	9.88	14.01	6.30	9.90	14.01	6.39	10.03	14.31	6.52	8.71	12.12	5.88	8.81	12.24	5.84	8.86	12.42	5.83	8.91	12.61	5.92	8.89	12.51	5.86
**Cannabis Use Disorder**																								
Global	52.81	72.96	40.00	50.76	68.03	38.86	49.39	65.66	37.68	48.30	65.15	36.80	48.25	65.78	36.98	47.03	62.51	36.14	47.02	62.54	36.04	48.78	65.76	37.09
Latin America and Caribbean	62.47	90.92	45.57	62.79	85.89	47.88	59.52	80.00	45.68	55.31	74.42	42.67	54.01	77.23	40.47	54.94	73.91	42.35	54.38	72.77	41.91	54.27	73.53	41.77
Argentina	40.55	54.22	30.30	41.90	51.52	33.60	40.96	50.25	32.97	38.95	48.14	31.17	39.60	52.98	29.80	40.06	49.05	32.32	40.07	49.06	32.33	40.67	50.54	32.61
Bolivia	42.53	57.91	31.74	46.51	62.07	35.67	47.18	62.86	36.28	43.00	56.88	33.25	39.24	52.12	29.71	41.13	53.96	31.94	41.30	54.12	32.07	41.33	54.15	32.09
Brazil	85.93	128.23	61.14	84.05	117.11	62.67	77.75	106.48	58.24	70.52	95.20	53.45	74.28	108.88	54.25	72.32	99.70	54.23	72.38	99.94	54.28	71.96	99.26	54.01
Chile	70.77	89.39	54.83	86.33	104.07	71.66	84.22	103.14	69.89	82.01	102.36	66.85	63.86	80.11	49.91	82.78	100.18	68.56	84.70	102.94	70.11	95.57	117.87	78.13
Colombia	57.41	76.25	43.10	84.51	105.91	67.27	82.44	103.14	65.71	79.93	101.69	63.05	49.84	65.21	38.17	74.98	93.47	59.87	75.42	94.08	60.18	80.99	103.34	63.84
Ecuador	53.48	74.14	38.94	51.65	69.99	37.89	50.56	68.04	37.20	49.63	66.44	36.71	46.31	62.67	34.57	45.80	60.77	34.17	45.90	60.92	34.22	46.04	61.14	34.32
Guyana	76.64	107.63	55.02	69.42	95.61	50.49	74.67	102.69	54.35	72.55	112.50	49.85	62.77	85.35	46.04	63.05	85.70	46.24	63.11	85.77	46.27	65.77	101.94	45.42
Paraguay	37.21	51.42	27.67	39.49	53.49	29.29	40.20	55.44	29.97	40.60	57.17	29.23	34.88	47.06	26.39	34.94	47.16	26.44	35.01	47.30	26.49	36.95	51.64	26.81
Peru	42.36	58.97	30.57	40.80	56.33	29.85	39.65	54.31	29.24	37.52	50.84	27.85	37.35	50.59	27.51	36.08	48.83	26.84	36.14	48.91	26.87	36.34	49.19	26.99
Suriname	78.70	123.98	53.20	73.96	107.65	53.03	68.96	99.19	49.78	64.76	92.68	46.99	68.56	105.88	47.30	65.27	93.84	47.20	65.10	93.71	47.07	65.13	93.83	47.07
Uruguay	76.15	103.79	54.86	77.78	98.06	61.78	76.68	96.63	60.88	69.20	86.92	54.26	78.11	106.31	56.30	81.55	102.95	64.77	81.56	102.94	64.78	79.34	100.25	62.11
Venezuela	40.77	59.73	28.56	38.12	56.32	26.61	38.29	56.14	26.72	33.97	47.99	24.39	36.19	51.53	26.01	34.09	49.38	24.05	34.26	49.61	24.18	33.99	49.18	24.02
**Cocaine Use Disorder**																								
Global	4.98	6.84	3.71	4.88	6.58	3.73	4.79	6.38	3.72	4.03	5.45	3.08	4.45	6.10	3.34	4.47	6.01	3.43	4.56	6.08	3.54	4.11	5.58	3.14
Latin America and Caribbean	12.78	19.05	8.67	13.77	20.17	9.44	15.77	22.71	11.10	12.84	18.81	8.97	10.46	15.38	7.28	11.54	16.65	8.02	14.13	20.30	10.00	12.82	18.85	8.94
Argentina	21.33	31.83	14.42	23.03	31.05	17.05	29.25	34.96	24.61	21.63	30.26	14.99	20.42	30.34	13.86	21.58	28.99	16.00	28.33	33.87	23.83	22.83	32.12	15.77
Bolivia	13.75	21.25	8.89	16.45	25.81	10.92	17.95	27.61	11.49	12.63	19.15	8.33	11.49	17.43	7.63	13.19	20.30	8.89	14.40	22.01	9.35	11.99	18.16	7.93
Brazil	12.64	18.71	8.63	15.95	23.67	10.80	17.68	26.07	12.24	14.35	21.07	10.15	10.65	15.51	7.47	13.14	19.26	9.00	16.67	24.48	11.46	15.35	22.68	10.74
Chile	18.58	27.17	12.88	21.53	27.77	16.77	22.77	30.34	17.17	15.67	23.14	10.79	16.34	23.78	11.39	20.80	26.86	16.22	22.61	30.17	16.99	18.72	28.09	12.60
Colombia	16.60	25.68	10.77	17.74	26.85	11.68	20.82	28.20	15.45	11.55	16.66	7.91	13.63	20.93	8.94	15.28	22.95	10.11	18.49	24.93	13.76	11.61	16.83	7.89
Ecuador	8.14	12.49	5.28	8.70	13.18	5.65	8.95	13.39	5.90	7.74	11.40	5.27	6.60	9.91	4.41	7.29	10.90	4.87	7.84	11.58	5.24	7.04	10.36	4.84
Guyana	11.92	17.77	7.85	10.48	16.21	7.03	11.95	18.15	7.96	10.66	15.79	7.01	9.23	13.58	6.23	9.47	14.55	6.37	9.57	14.31	6.55	9.58	14.23	6.28
Paraguay	3.95	5.86	2.64	4.64	7.10	3.09	4.67	7.01	3.16	4.00	5.78	2.77	3.53	5.11	2.43	3.81	5.68	2.62	3.86	5.71	2.64	3.62	5.23	2.51
Peru	9.27	13.94	6.12	9.61	14.48	6.41	9.51	14.07	6.41	7.69	11.59	5.19	7.56	11.14	5.15	7.97	11.98	5.40	8.41	12.40	5.75	7.57	11.46	5.07
Suriname	12.22	18.61	8.17	13.68	20.87	9.10	11.92	18.32	8.15	11.03	16.80	7.43	10.26	15.49	6.98	11.67	17.69	7.78	11.44	17.58	7.79	11.18	17.07	7.52
Uruguay	21.03	31.79	13.97	21.19	30.56	14.62	26.18	36.35	19.54	20.72	32.14	13.95	21.39	32.28	14.23	22.10	31.92	15.20	27.71	38.54	20.67	23.81	37.08	15.95
Venezuela	3.93	5.90	2.66	4.08	6.07	2.77	4.19	6.12	2.88	3.41	4.94	2.36	3.29	4.86	2.26	3.49	5.12	2.40	3.67	5.33	2.53	3.47	5.09	2.39
**Opioid Use Disorder**																								
Global	34.17	45.23	26.23	37.09	48.28	29.01	39.29	50.94	30.70	39.86	51.73	31.46	32.64	42.64	25.49	35.78	46.04	28.21	37.23	48.20	29.29	39.22	50.65	30.88
Latin America and Caribbean	21.39	28.57	16.28	22.92	30.30	17.65	24.60	32.00	19.29	26.41	33.67	20.83	21.28	27.41	16.54	22.31	28.60	17.50	23.34	29.97	18.38	25.06	32.02	19.79
Argentina	12.41	16.40	9.57	14.71	18.88	11.40	15.73	20.21	12.24	17.17	22.04	13.43	12.77	16.80	9.86	14.46	18.36	11.30	15.08	19.29	11.71	16.37	21.21	12.67
Bolivia	28.15	38.20	21.01	30.03	40.28	22.47	26.38	34.38	20.22	26.58	34.63	20.78	30.80	40.07	23.74	31.43	40.65	24.27	26.37	33.65	20.73	27.01	34.71	21.45
Brazil	23.06	30.67	17.44	25.06	33.26	19.21	28.62	37.12	22.17	31.95	40.67	25.02	22.59	29.16	17.41	23.64	30.64	18.30	26.42	34.00	20.61	30.32	39.12	23.57
Chile	16.25	20.56	12.63	14.65	19.00	11.38	15.35	19.98	11.72	16.34	21.14	12.67	15.31	19.00	12.21	14.30	18.61	11.12	14.23	18.59	10.83	15.58	20.65	11.94
Colombia	23.67	31.70	17.71	22.55	29.93	17.16	23.35	31.03	18.06	25.23	33.02	19.42	22.80	29.87	17.54	22.23	28.86	17.19	22.51	29.65	17.44	23.51	30.83	18.09
Ecuador	18.58	25.17	14.04	21.65	28.54	16.35	25.32	32.88	19.68	27.19	35.10	21.28	19.63	25.67	15.29	22.05	28.09	17.10	25.38	32.54	19.98	26.35	33.51	20.76
Guyana	17.94	23.74	13.65	20.82	26.14	16.48	20.04	25.14	15.71	23.26	29.02	18.34	17.60	22.27	13.80	20.66	25.60	16.59	19.88	24.83	15.80	21.32	26.09	16.99
Paraguay	20.32	27.35	15.20	20.96	28.19	15.47	21.35	28.98	16.01	21.57	28.49	16.27	21.90	28.45	16.75	21.62	28.52	16.26	20.28	26.81	15.56	20.22	26.33	15.46
Peru	19.84	25.82	15.19	22.95	29.61	17.67	27.70	35.28	21.40	27.58	34.74	21.62	20.26	25.65	15.92	22.57	28.16	17.82	26.43	33.28	20.70	26.57	33.38	21.00
Suriname	15.38	20.34	11.53	17.90	22.80	14.11	19.16	24.44	15.02	20.28	25.62	16.12	14.90	18.94	11.45	17.09	21.50	13.61	18.37	23.30	14.48	19.90	25.27	15.77
Uruguay	16.87	22.44	12.64	18.38	24.45	14.11	17.83	23.19	13.78	18.44	24.02	14.29	16.93	22.72	12.57	18.13	24.16	13.79	17.36	22.90	13.31	17.79	23.56	13.51
Venezuela	27.65	37.40	20.45	28.55	38.12	21.47	27.52	36.39	21.04	25.03	32.23	19.78	27.32	35.53	20.82	27.48	35.69	21.16	25.55	33.51	19.84	24.83	32.01	19.49

Table S9. Estimates of the South American Population, Global Burden of Disease Study, 1990-2019.

Year	Gender				Age (in years)										Total
	Male		Female		0-19		20-39		40-59		60-79		80 plus		
	n	%	n	%	n	%	n	%	n	%	n	%	n	%	
1990	144.9	49.4%	148.3	50.6%	132.9	45.3%	91.7	31.3%	46.3	15.8%	19.7	6.7%	2.6	0.9%	293.1
1991	147.4	49.4%	151.0	50.6%	134.3	45.0%	93.5	31.3%	47.6	16.0%	20.3	6.8%	2.7	0.9%	298.4
1992	149.9	49.4%	153.7	50.6%	135.6	44.6%	95.3	31.4%	49.0	16.1%	20.9	6.9%	2.8	0.9%	303.7
1993	152.5	49.4%	156.4	50.6%	136.8	44.3%	97.1	31.4%	50.6	16.4%	21.5	7.0%	3.0	1.0%	308.9
1994	155.0	49.3%	159.1	50.7%	137.9	43.9%	98.9	31.5%	52.2	16.6%	22.1	7.0%	3.1	1.0%	314.1
1995	157.5	49.3%	161.8	50.7%	138.8	43.5%	100.6	31.5%	53.9	16.9%	22.8	7.1%	3.3	1.0%	319.3
1996	160.1	49.3%	164.5	50.7%	139.7	43.1%	102.4	31.6%	55.6	17.1%	23.4	7.2%	3.4	1.0%	324.5
1997	162.6	49.3%	167.1	50.7%	140.4	42.6%	104.2	31.6%	57.5	17.4%	24.1	7.3%	3.6	1.1%	329.7
1998	165.2	49.3%	169.8	50.7%	141.0	42.1%	106.0	31.7%	59.4	17.7%	24.8	7.4%	3.7	1.1%	334.9
1999	167.7	49.3%	172.4	50.7%	141.5	41.6%	107.8	31.7%	61.4	18.1%	25.6	7.5%	3.9	1.1%	340.1
2000	170.3	49.3%	175.0	50.7%	141.8	41.1%	109.6	31.7%	63.4	18.4%	26.3	7.6%	4.1	1.2%	345.2
2001	172.8	49.3%	177.5	50.7%	141.9	40.5%	111.4	31.8%	65.6	18.7%	27.1	7.7%	4.2	1.2%	350.3
2002	175.2	49.3%	180.0	50.7%	142.0	40.0%	113.2	31.9%	67.8	19.1%	27.9	7.9%	4.4	1.2%	355.3
2003	177.6	49.3%	182.5	50.7%	141.9	39.4%	115.0	31.9%	70.0	19.4%	28.7	8.0%	4.6	1.3%	360.2
2004	180.1	49.3%	185.0	50.7%	141.9	38.9%	116.6	31.9%	72.2	19.8%	29.5	8.1%	4.8	1.3%	365.1
2005	182.4	49.3%	187.5	50.7%	141.8	38.3%	118.3	32.0%	74.5	20.1%	30.4	8.2%	5.1	1.4%	370.0
2006	184.8	49.3%	190.0	50.7%	141.6	37.8%	119.9	32.0%	76.7	20.5%	31.2	8.3%	5.3	1.4%	374.7
2007	187.0	49.3%	192.2	50.6%	141.3	37.2%	121.6	32.0%	78.6	20.7%	32.3	8.5%	5.6	1.5%	379.5
2008	189.3	49.3%	194.9	50.7%	141.0	36.7%	123.2	32.1%	80.8	21.0%	33.3	8.7%	5.9	1.5%	384.1
2009	191.5	49.3%	197.3	50.7%	140.7	36.2%	124.8	32.1%	82.7	21.3%	34.5	8.9%	6.1	1.6%	388.8
2010	193.7	49.2%	199.7	50.8%	140.3	35.7%	126.3	32.1%	84.7	21.5%	35.7	9.1%	6.4	1.6%	393.4
2011	195.9	49.2%	202.1	50.8%	139.9	35.2%	128.0	32.2%	86.6	21.8%	36.9	9.3%	6.7	1.7%	398.0
2012	198.1	49.2%	204.6	50.8%	139.6	34.7%	129.5	32.2%	88.4	22.0%	38.3	9.5%	6.9	1.7%	402.7
2013	200.4	49.2%	207.1	50.8%	139.3	34.2%	131.1	32.2%	90.3	22.2%	39.7	9.7%	7.2	1.8%	407.5
2014	202.6	49.2%	209.6	50.8%	139.0	33.7%	132.5	32.1%	92.1	22.3%	41.2	10.0%	7.4	1.8%	412.2
2015	204.7	49.1%	211.9	50.9%	138.5	33.2%	133.7	32.1%	94.0	22.6%	42.7	10.2%	7.7	1.8%	416.6
2016	206.5	49.1%	213.9	50.9%	137.8	32.8%	134.6	32.0%	95.8	22.8%	44.3	10.5%	8.0	1.9%	420.4
2017	208.1	49.1%	215.8	50.9%	136.9	32.3%	135.3	31.9%	97.6	23.0%	45.9	10.8%	8.3	2.0%	423.9
2018	209.8	49.1%	217.7	50.9%	136.0	31.8%	135.9	31.8%	99.5	23.3%	47.6	11.1%	8.6	2.0%	427.5
2019	211.5	49.1%	219.7	51.0%	135.2	31.4%	136.4	31.6%	101.3	23.5%	49.3	11.4%	8.9	2.1%	431.1
Average	180.2	49.3%	185.6	50.7%	139.2	38.6%	116.5	31.8%	73.2	19.7%	31.6	8.5%	5.3	1.4%	365.8
